# One-pot sequential synthesis of isocyanates and urea derivatives via a microwave-assisted Staudinger–aza-Wittig reaction

**DOI:** 10.3762/bjoc.9.274

**Published:** 2013-11-06

**Authors:** Diego Carnaroglio, Katia Martina, Giovanni Palmisano, Andrea Penoni, Claudia Domini, Giancarlo Cravotto

**Affiliations:** 1 Dipartimento di Scienza e Tecnologia del Farmaco, University of Turin, Via Pietro Giuria 9, 10125 Torino, Italy; Fax +390116707687; Tel: +390116707684; 2 Dipartimento di Scienza e Alta Tecnologia, University of Insubria, Via Valleggio 11, 22100 Como, Italy; 3 Department of Chemistry, Universidad Nacional del Sur, Av. Alem 1253, B8000CPB, Bahía Blanca, Buenos Aires, Argentina

**Keywords:** isocyanates, microwave-assisted reaction, one-pot reaction, tandem Staudinger–aza-Wittig reaction, urea derivatives

## Abstract

A fast and efficient protocol for the synthesis of *N,N'*-disubstituted urea derivatives from alkyl halides and primary or secondary amines has been developed. The synthetic pathway combines nucleophilic substitutions and a Staudinger–aza-Wittig reaction in the presence of polymer-bound diphenylphosphine under 14 bar of CO_2_ pressure and has been performed in a one-pot two-step process. The protocol has been optimized under microwave irradiation and the scale-up experiment has been conducted under conventional conditions in a Parr reactor. The final compounds were isolated after simple filtration in almost quantitative overall yields which makes this procedure facile and rapid to execute.

## Introduction

The industrial and commercial impact of isocyanates (R–NCO) is steadily growing. In particular, the polyurethane output has undergone yearly increases of 5% over the last decade [1]. Isocyanates play a relevant role as chemical intermediates in the manufacturing of thermoplastic foams, elastomers, adhesives, agrochemicals and pharmaceuticals. The isocyanate group is also widely used as a precursor to several bioactive compounds and drugs that contain urea and carbamate moieties [2]. Isocyanates were discovered by Wurtz in 1849 [3], and more than 20 methods for the preparation of R–NCO have now been listed and classified according to the reactions involved [4–5]. The old procedure that entails reactions between primary or secondary amines or amides with phosgene, is the most commonly used and it is described in papers [6–7] and reviews [8]. The main drawbacks of this method are the extremely high toxicity of phosgene and the generation of a large amount of corrosive HCl. Moreover, the high temperature required by this process (>250 °C) makes the synthesis of lower molecular weight compounds impossible.

Apart from the catalytic carbonylation of nitro compounds, which is one of the most interesting alternatives for the synthesis of aromatic isocyanates, other alternative greener, non-phosgene routes to isocyanates have also been developed [9]. Of these, the Curtius, Hoffman and Lossen rearrangements have been used quite often in the past and are still used for specific applications [10–13]. The Staudinger–aza-Wittig reactions have played a pivotal role in the construction of cyclic and acyclic compounds [14–19]. The replacement of phosgene by carbon dioxide (CO_2_), which is nontoxic, abundant, and economical, is the main advantage of this reaction. The mechanism of this transformation passes through iminophosphoranes that are versatile intermediates and can react with CO_2_ to generate isocyanates [20]. This reaction is compatible with a large number of functional groups and therefore has various uses in organic synthesis and can also be exploited for the preparation of heterocyclic compounds. Isocyanate derivatives can be generally obtained in good yields. However, it is necessary to avoid the traditional triphenylphosphine to obtain high purity products [21].

So called “enabling techniques”, mainly non-conventional energy sources such as microwaves (MW) and ultrasound (US), can dramatically enhance reaction rates in organic synthesis [22–23]. Kinetics and yields of any chemical modification are strongly improved by the optimal heat and mass transfer provided by dielectric heating and sonochemical conditions [24–25]. In general, MW in organic synthesis is a valid response to problems regarding long reaction times and a high reagent excess. The use of dielectric heating to promote chemical reactions has become well established as a reliable technique which can be applied on a range of scales [26]. Despite MW irradiation being commonly used in organic synthesis, only few publications describe this technique with gaseous reagents in closed vessels and in heterogeneous gas-phase reactions that are important for industrial processes [27–31]. The aim of the present work is the development of new green and efficient synthetic procedures for easier access to isocyanates and urea libraries using a renewable carbon resource like CO_2_. Since CO_2_ requires a large energy input to be transformed [32], we have studied a synthetic procedure which uses a MW reactor (SynthWave by Milestone) and is well suited for parallel syntheses at any reaction temperature and gas pressure (up to 300 °C and 200 bar). We aimed to successfully reduce the reaction time and the reagent excess, and to employ poorly reactive substrates and volatile, solid and supported reagents. In summary, we herein report an optimized protocol for a MW-assisted Staudinger–aza-Wittig reaction with polymer-bound diphenylphosphine (PS-PPh_2_) in a CO_2_ atmosphere. The study also aimed to prepare a series of symmetric and asymmetric alkyl/aryl urea derivatives in a one-pot, sequential synthesis of urea derivatives from alkyl bromides. With the aim to verify the feasibility of the method under conventional heating, the protocol was tested in a Parr reactor (90 mL) for an easier scale-up.

## Results and Discussion

**Scheme 1:**
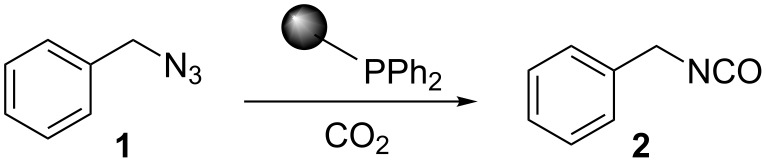
Synthesis of benzyl isocyanate.

The Staudinger–aza-Wittig reaction is extremely versatile and can be used for the synthesis of many products. However, the byproduct of this reaction is triphenylphosphine oxide, which is difficult to remove. It is known that this reaction can be performed in a heterogeneous system using PS-PPh_2_. Despite the higher costs for the reagent, the use of PS-PPh_2_ has the advantage of a much easier reaction work-up [33–38]. A polymer regeneration procedure was described by Marsura et al. [35], however a quasi-stoichiometric amount makes the recycling step unneccessary. With the aim to overcome cost limitations we recently described the preparation of triphenylphosphine-loaded cross-liked cyclodextrin complexes as recyclable green catalyst [39]. The reactivity of polystyrene-supported reagents strongly depends on the choice of solvent that can influence polymer swelling [40]. Solvent choice is therefore an important issue as it must allow the supported reagent to work in a friendly environment and, at the same time, facilitate the reaction outcome. In the first part of this work, we have focused on the development of a MW promoted protocol for isocyanate synthesis with the aim of reducing reaction time and decreasing the amount of solid supported PS-PPh_2_, which is usually added in large excess. The conversion of benzyl azide to benzyl isocyanate (Scheme 1) was selected as the model reaction and it was performed both under conventional conditions and under MW irradiation. Various solvents were compared at a number of temperatures thanks to the versatility of the SynthWave reactor, which provides multiple-sample racks. Experiments were performed at 90, 70 and 50 °C at 14.5 bar of CO_2_ pressure.

As shown in Table 1, the reaction showed complete conversion when performed in toluene at 70 °C. The purity of the compound was also slightly higher than that of the reacition at 90 °C. The conversion was not complete at 50 °C. Conversion was low in THF and the isocyanate was present only as trace among side-products. MeCN, like toluene allowed a higher conversion and a higher yield to be achieved, even in comparison with DMF. In MeCN, there was a complete conversion even at 50 °C in 4 h. The influence of the CO_2_ pressure on the reaction rate was evident by the poor conversion (40%) that was observed when the reaction was performed with 1 bar CO_2_ at 50 °C in an oil bath. In contrast, the conversion reached 93% in a Parr reactor with 14 bar CO_2_. At room temperature with 1 bar CO_2_ the reaction occurs within 24 h.

**Table 1:** Synthesis of benzyl isocyanate.^a^

entry	solvent	reaction conditions	conversion^b^(%)	yield^b^(%)

1	toluene	90 °C, MW, CO_2_ (14.5 bar)	>99	75
2	toluene	70 °C, MW, CO_2_ (14.5 bar)	96	84
6	toluene	50 °C, MW, CO_2_ (14.5 bar)	82	78
3	THF	70 °C, MW, CO_2_ (14.5 bar)	50	25
4	DMF	70 °C, MW, CO_2_ (14.5 bar)	95	80
5	MeCN	70 °C, MW, CO_2_ (14.5 bar)	>99	85
7	MeCN	50 °C, MW, CO_2_ (14.5 bar)	>99	94
8^c^	MeCN	50 °C, CO_2_ (1 bar)	41	25
9	MeCN	rt, CO_2_ (1 bar)	25 (95)^d^	21 (85)^d^
10^e^	MeCN	50 °C, CO_2_ (14 bar)	93	89

^a^Unless otherwise stated, reactions were performed in the presence of PS-PPh_2_ (5 equiv), reaction time 4 h. ^b^Determined by GC–MS. ^c^The reaction was performed in an oil bath. ^d^Reaction time 24 h. ^e^The reaction was performed in a Parr reactor.

In order to optimize the reaction conditions, another study was pursued in MeCN and toluene, and a number of different reactions were performed at 50 °C. As shown in Table 2, we confirm that the reaction was faster in MeCN than in toluene and that even full conversion was obtained after 1.5 h in many cases. An important goal was to reduce the PS-PPh_2_ excess from 5 to 1.5 equiv, with excellent results only in MeCN (Table 2) under MW irradiation. When the reaction was carried out in the Parr reactor, the highest conversion was 93% with 2 equiv PS-PPh_2_.

**Table 2:** Synthesis of benzyl isocyanate.^a^

entry	solvent	PS-PPh_2_ (equiv)	time (h)	conversion^b^ (%)	yield^b^ (%)

1	toluene	5	4	82	78
2	toluene	5	2	71	71
3	toluene	2	2	66	54
4	MeCN	5	4	>99	94
5	MeCN	5	2	>99	96
6	MeCN	5	1.5	>99	97
7	MeCN	5	1	80	76
8	MeCN	3	1.5	>99	97
9	MeCN	2	1.5	>99	95
10^c^	MeCN	2	1.5	93	88
11	MeCN	1.5	1.5	>99	98
12^c^	MeCN	1.5	1.5	88	82
13	MeCN	1	1.5	80	76

^a^Reactions were carried out in a MW reactor: **1**, PS-PPh_2_, CO_2_ (14.5 bar) 50 °C. ^b^Determined by GC–MS. ^c^Reactions were carried out in a Parr reactor (90 mL): **1**, PS-PPh_2_, CO_2_ (14 bar) 50 °C.

To confirm the versatility of our protocol, the method was extended to include a number of different substrates and the obtained isocyanates were also used for the synthesis of urea derivatives via the reaction with (±)-1-phenylethylamine **3** (see Scheme in Table 3). Seven different azido-derivatives were synthesized from the alkyl halide and reacted via Staudinger–aza-Wittig reactions. After the PS-PPh_2_ was filtered, the isocyanate solution was directly subjected to an addition of 2 equiv of **3** and heated up to 70 °C in the MW reactor for 3 h. Primary alkyl and benzyl azide derivatives were compared to secondary ones, and the compatibility of the protocol towards different functional groups was also considered. The results obtained are given in Table 3 and show the yield of urea derivatives after purification from the amine excess by using Dowex^®^ 50WX8-200. This synthetic protocol is versatile and highly efficient with both primary and secondary azido derivatives as well as alkoxy and amido groups. The one-pot, two-step procedure afforded urea derivatives in high yield and purity via the isocyanate intermediate. To broaden the scope of the study, the reaction was repeated in a bigger scale (80 mL) in a Parr reactor at the same pressure. Despite the good conversion (about 93%), the product purity was slightly lower than that of the MW-assisted reaction.

**Table 3:** Synthesis optimization of urea derivatives.^a^

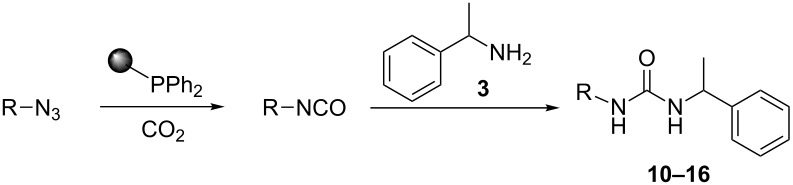			

entry	R–N_3_	product	yield^b^ (%)

1	**1**	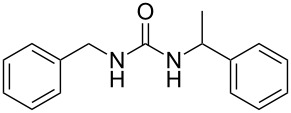 **10**	98
1^c^	**1**	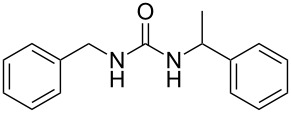 **10**	79
2	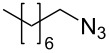 **4**	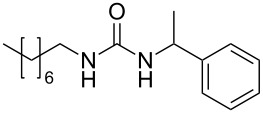 **11**	90
3	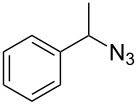 **5**	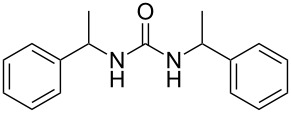 **12**	92
4	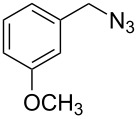 **6**	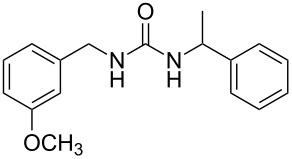 **13**	97
5	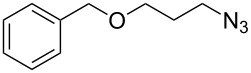 **7**	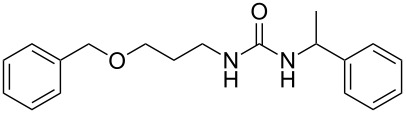 **14**	97
6	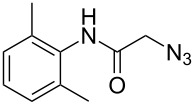 **8**	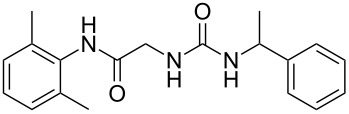 **15**	98
7	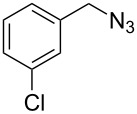 **9**	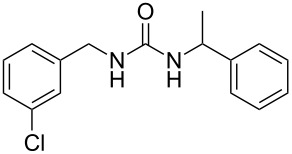 **16**	94

^a^Reactions were carried out in a MW reactor: azido derivative, PS-PPh_2_ (1.5 equiv), CO_2_ (14.5 bar), 50 °C, 1.5 h, then **3** (2 equiv) 70 °C, 3 h. ^b^Isolated yield. ^c^The reaction was performed in a Parr reactor.

To expand the scope of this method, a sequential one-pot synthesis from the alkyl bromide to the urea derivative was carried out without isolating the intermediates. The aim of this part of the work was the synthesis of the azido derivatives and their subsequent conversion to urea via the Staudinger–aza-Wittig reaction and one-pot amine addition. The combination of reactants in a one-pot fashion can lead to undesired side-product formation and, consequently, a lower yield. Therefore, the choice of the right solvent and reaction conditions is the key to the success of this transformation.

Our initial attempts focused on the synthesis of azido derivatives in MeCN. Although generally performed in DMF, the S_N_2 reaction of alkyl bromide with NaN_3_ can also be performed in MeCN. NaN_3_ is insoluble in MeCN at room temperature (<0.005 g/100 mL), but its solubility increases at higher temperatures. Furthermore, NaBr generated during the nucleophilic substitution is insoluble and can be removed by filtration as can the NaN_3_ excess. These factors allow the work-up procedure to be simplified and pure azido derivative solutions were obtained by filtration. Benzyl azide was successfully obtained from benzyl bromide after the reaction in a MW reactor at 95 °C for 3 h. After filtration, the benzyl azide solution was directly converted into the urea compound by MW irradiation at 70 °C for 3 h in the presence of CO_2_, PS-PPh_2_ and benzylamine. The desired product was obtained in almost quantitative yields.

The robustness of this MW-promoted sequential one-pot procedure was established by synthesizing a set of 13 different urea derivatives. A small set of five different primary and secondary alkyl and benzyl azides were synthesized. Besides the azido derivatives synthesized from **17**–**19**, the two volatile azides synthesized from **20** and **21** (*n*-butyl and allyl azide, respectively) were obtained under N_2_ pressure. After filtration they were converted into the urea compound. The procedure was performed in parallel and under 14.5 bar of CO_2_ and even the volatile allyl and butyl azide reacted successfully. The results reported in Table 4 show that all final products were obtained in excellent to almost quantitative yield.

**Table 4:** One-pot MW-assisted synthesis of a set of urea derivatives.^a^

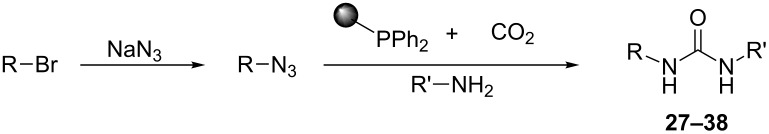				

entry	R–Br	R–NH_2_	product	yield^b^ (%)

1	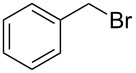 **17**	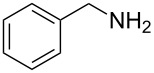 **22**	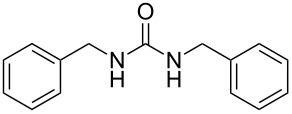 **27**	98
2	**17**	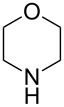 **23**	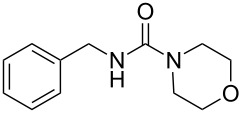 **28**	98
3	**17**	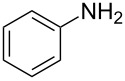 **24**	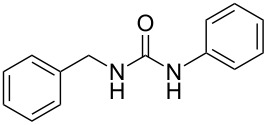 **29**	98
4	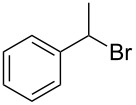 **18**	**22**	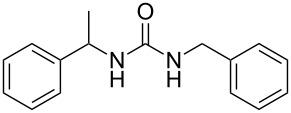 **10**	97
5	**18**	**23**	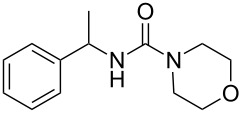 **30**	94
6	**18**	**24**	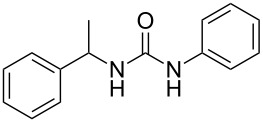 **31**	98
7	**18**	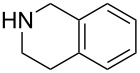 **25**	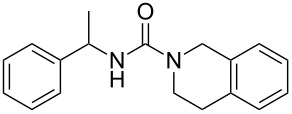 **32**	98
8	**18**	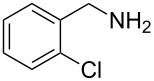 **26**	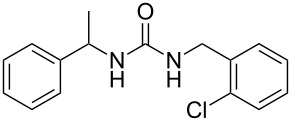 **33**	98
9	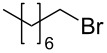 **19**	**22**	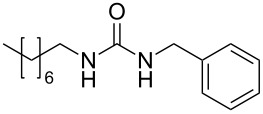 **34**	89
10	**19**	**23**	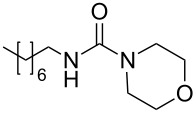 **35**	89
11	**19**	**24**	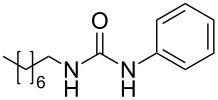 **36**	88
12^c^	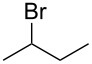 **20**	**3**	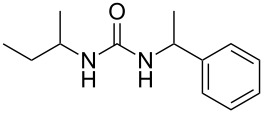 **37**	85
13^c^	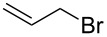 **21**	**3**	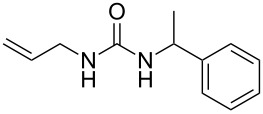 **38**	83

^a^Reactions were carried out in a MW reactor: alkyl bromide, NaN_3_ (2 equiv), MeCN, 95 °C, 3 h ; then PS-PPh_2_ (1.5 equiv), CO_2_ (14.5 bar), amine (2 equiv), at 50 °C 1.5 h then 70 °C 3 h. ^b^Isolated yield. ^c^R–N_3_ was synthesized in DMF and MeCN was then added.

## Conclusion

In conclusion, a MW-assisted, one-pot sequential protocol for the synthesis of urea derivatives from alkyl bromides has been described. This study has proven that in acetonitrile under high CO_2_ pressure the Staudinger–aza-Wittig reaction in presence of PS-PPh_2_ is strongly promoted. Excellent results have been obtained under MW irradiation in a closed vessel also with gaseous reagents. The optimized procedure benefited from the use of a quasi-stoichiometric amount of PS-PPh_2_ and can be applied for the efficient, safe, rapid, and cost-effective production of urea derivative libraries.

## Experimental

All chemicals were purchased from Sigma-Aldrich (solvents from Carlo Erba SpA) and used without further purification. Solid diphenylphosphino-polystyrene (PS-PPh_2_) was purchased from Novabiochem^®^ (Cas-No: 39319-11-4, loading ca. 1.2 mmol/g). Reactions were monitored by TLC on Merck 60 F_254_ (0.25 mm) plates, which were visualized by UV inspection and/or by heating after a spraying with 5% H_2_SO_4_ in ethanol or phosphomolybdic acid. MW-promoted reactions were carried out in a SynthWave (Milestone, Italy). NMR spectra were recorded on a Bruker Avance 300 (300 MHz and 75 MHz for ^1^H and ^13^C, respectively) at 25 °C; chemical shifts were calibrated to the residual proton and carbon resonances of the solvents: CDCl_3_ (δH = 7.26, δC = 77.16) or CD_3_OD (δH = 3.31, δC = 49.00). GC–MS analyses were performed in a GC Agilent 6890 (Agilent Technologies, USA) that was fitted with a mass detector Agilent Network 5973, using a 30 m long capillary column, i.d. of 0.25 mm and film thickness 0.25 μm. GC conditions were: injection split 1:20, injector temperature 250 °C, detector temperature 280 °C. Gas carrier: helium (1.2 mL/min), temperature program: from 70 °C (2 min) to 300 °C at 5 °C/min. HRMS was determined using MALDI-TOF mass spectra (Bruker Ultraflex TOF mass spectrometer).

### General procedures

**Representative procedure for alkyl isocyanate synthesis from alkyl azide:** PS-PPh_2_ (0.477 mmol) was added to a solution of alkyl azide (0.318 mmol) in MeCN (1.5 mL). The mixture was irradiated by MW for 1.5 h at 50 °C (average power 70 W) under CO_2_ (14.5 bar) and magnetic stirring. After the reaction, the mixture was filtered on a cartridge. When the reaction was performed in a Parr reactor, PS-PPh_2_ (6.36 mmol) was added to a solution of alkyl azide (3.18 mmol) in MeCN (80 mL). The solution was heated for 2 h at 50 °C under CO_2_ (14.5 bar). After the reaction, the mixture was filtered on a cartridge.

**Representative procedure for urea synthesis from alkyl isocyanate:** The amine (0.636 mmol) was added to a solution of alkyl isocyanate (0.318 mmol) in MeCN (1.5 mL). The solution was irradiated by MW for 3 h at 70 °C (average power 200 W) under N_2_ (2 bar) and magnetic stirring. The solvent was then evaporated under vacuum, the residue was dissolved in MeOH and Dowex^®^ 50WX8-200 was added. The mixture was stirred at rt for 15 min. The mixture was then filtered on paper and the solvent was evaporated under vacuum.

**Representative “multi-pot” procedure for the synthesis of urea derivatives:** NaN_3_ (0.477 mmol) was added to a solution of alkyl bromide (0.318 mmol) in MeCN (1.5 mL). The mixture was irradiated by MW for 3 h at 95 °C (average power 240 W) under N_2_ (2 bar) and magnetic stirring. After the reaction, the mixture was cooled to rt, filtered on paper, and PS-PPh_2_ (0.477 mmol) and amine (0.636 mmol) were sequentially added. The mixture was irradiated by MW for 1.5 h at 50 °C (average power 70 W) and 3 h at 70 °C (average power 200 W) under CO_2_ (14.5 bar) and magnetic stirring. Then, the mixture was filtered on a cartridge to remove the polymer-bound diphenylphosphine oxide. The solvent was then evaporated under vacuum, the residue was dissolved in MeOH and Dowex^®^ 50WX8-200 was added. The mixture was stirred at rt for 15 min. Finally, the mixture was filtered on paper and the solvent was evaporated under vacuum. When the reaction was performed in a Parr reactor, PS-PPh_2_ (6.36 mmol) was added to a solution of alkyl azide (3.18 mmol) and amine (6.36 mmol) in MeCN (80 mL). The solution was heated 3 h at 70 °C under CO_2_ (14.5 bar). The mixture was filtered on a cartridge to remove the polymer-bound diphenylphosphine oxide and the residual polymer-bound diphenylphosphine. The solvent was then evaporated under vacuum, the residue was dissolved in MeOH and Dowex^®^ 50WX8-200 was added. The mixture was stirred at rt for 15 min. Finally, the mixture was filtered on paper and the solvent was evaporated under vacuum.

## Supporting Information

File 1 Detailed analytical data of the prepared compounds and a collection of NMR spectra.
