# Supplementary material for: One-pot sequential synthesis of isocyanates and urea derivatives via a microwave-assisted Staudinger–aza-Wittig reaction
Source: Beilstein J Org Chem. 2013 Nov 6;9:2378–86. doi: 10.3762/bjoc.9.274 (PMC3869261; doi:10.3762/bjoc.9.274)
Supplement: File 1 — Detailed analytical data of the prepared compounds and a collection of NMR spectra. [file Beilstein_J_Org_Chem-09-2378-s001.pdf]

Supporting Information

for

One-pot sequential synthesis of isocyanates and urea compounds via a microwave-assisted Staudinger–aza-Wittig reaction

Diego Carnaroglio[1], Katia Martina[1], Giovanni Palmisano[2], Andrea Penoni[2], Claudia Domini[3] and Giancarlo Cravotto*[1]

Address: [1] Dipartimento di Scienza e Tecnologia del Farmaco, University of Turin, Via Pietro Giuria 9, 10125 Torino, Italy; Fax +390116707687; Tel: +390116707684; [2]Dipartimento di Scienza e Alta Tecnologia, University of Insubria, Via Valleggio 11, 22100 Como, Italy and [3] Department of Chemistry, Universidad Nacional del Sur, Av. Alem 1253, B8000CPB, Bahía Blanca, Buenos Aires, Argentina

Email: Giancarlo Cravotto* - giancarlo.cravotto@unito.it

* Corresponding author

Detailed analytical data of the prepared compounds and a collection of NMR spectra

Table of contents

1. Analytical Data…………………….. S3
2. ^{1}H and ^{13}C NMR spectra ………………………………………………………………. S8

1. Analytical Data

***N*-benzyl-*N′*-(1-phenylethyl)urea 10 (Table 3, entry 1)** [1]:

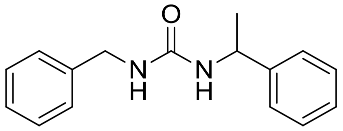

Yield 79 mg (98%); a white solid. ^{1}H NMR (300 MHz, $CDCl_3$) δ 7.37 – 7.13 (m, 10H), 4.76 (q, *J*=6.00 Hz, 1H), 4.33 (s, 2H), 1.46 (d, *J*=6 Hz, 3H) ppm. ^{13}C NMR (75 MHz, $CDCl_3$) δ 158.13, 144.16, 139.07, 129.09, 128.91, 127.68, 127.60, 127.53, 126.16, 50.83, 44.69, 23.71 ppm. *m/z* (MALDI-TOF MS): calcd for $C_{16}H_{18}N_2O$ $[M + H]^+$: 255.1419, found: 255.1416.

***N*-Octyl-*N′*-(1-phenylethyl)urea 11 (Table 3, entry 2):**

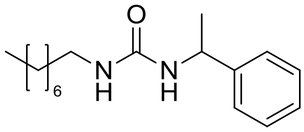

Yield 79 mg (90%); a yellowish oil. ^{1}H NMR (300 MHz, $CDCl_3$) δ 7.36 – 7.26 (m, 5H), 4.71 (q, *J*=6 Hz, 1H), 3.11 (t, *J*=6 Hz, 2H), 1.48 (m, 2H), 1.32 – 1.09 (o.s., 10H), 0.87 (o.s., 6H) ppm. ^{13}C NMR (75 MHz, $CDCl_3$) δ 159.35, 137.62, 129.26, 128.25, 127.75, 45.25, 41.53, 32.09, 29.84, 29.49, 29.48, 27.00, 22.97, 14.44 ppm. *m/z* (MALDI-TOF MS): calcd for $C_{17}H_{28}N_2O$ $[M + H]^+$: 277.2201, found: 277.2198.

***N,N′*-bis(1-phenylethyl)urea 12 (Table 3, entry 3)** [2]:

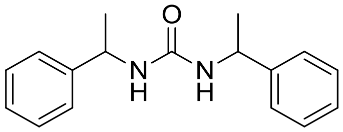

Yield 78 mg (92%); a white solid. ^{1}H NMR (300 MHz, $CDCl_3$) δ 7.36 – 7.09 (m, 10H), 4.73 (q, *J*=6 Hz, 2H), 1.34 (d, *J*=6 Hz, 6H) ppm. ^{13}C NMR (300 MHz, $CDCl_3$) δ 157.57, 144.13, 129.02, 127.57, 126.05, 50.74, 23.69 ppm. *m/z* (MALDI-TOF MS): calcd for $C_{17}H_{20}N_2O$ $[M + H]^+$: 269.1576, found: 269.1569.

***N*-(3-methoxybenzyl)-*N′*-(1-phenylethyl)urea 13 (Table 3, entry 4):**

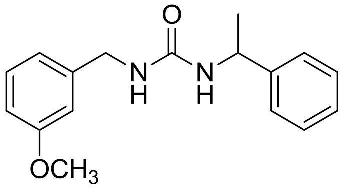

Yield 87 mg (97%); a withe solid. ^{1}H NMR (75 MHz, CD_3OD) δ 7.34 – 7.13 (m, 5H), 6.87 – 6.72 (m, 4H), 4.84 (q, *J*=6 Hz, 1H), 4.27 (s, 2H), 3.74 (s, 3H), 1.42 (d, *J*=9 Hz, 3H) ppm. ^{13}C NMR (75

MHz, CD_3OD) δ 161.33, 160.30, 146.43, 142.89, 130.45, 129.47, 127.85, 126.81, 120.30, 113.52, 113.46, 64.30, 55.56, 44.56, 23.57 ppm. *m/z* (MALDI-TOF MS): calcd for $C_{17}H_{20}N_2O_2$ $[M + H]^+$: 285.1524, found: 285.1527.

***N*-(3-(benzyloxy)propyl)-*N′*-(1-phenylethyl)urea 14 (Table 3, entry 5):**

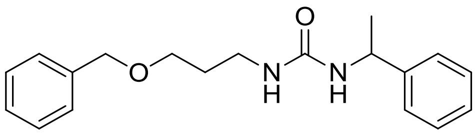

Yield 96 mg (97%); a yellowish oil. ^1H NMR (300 MHz, $CDCl_3$) δ 7.35 – 7.15 (m, 10H), 4.50 (q, 1H), 4.38 (s, 2H), 3.48 (t, 2H), 3.26 (t, 2H), 1.72 (m, 2H), 1.38 (d, J=6 Hz, 3H) ppm. ^{13}C NMR (75 MHz, $CDCl_3$) δ 158.93, 143.28, 138.14, 128.98, 128.73, 128.09, 127.72, 126.02, 73.31, 63.97, 51.33, 39.04, 29.51, 23.46 ppm. *m/z* (MALDI-TOF MS): calcd for $C_{19}H_{24}N_2O_2$ $[M + H]^+$: 313.1838, found: 313.1836.

***N*-(2,6-dimethylphenyl)-2-(3-(1-phenylethyl)ureido)acetamide 15 (Table 3, entry 6):**

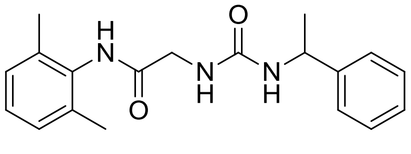

Yield 100 mg (98%); a white solid. ^1H NMR (300 MHz, CD_3OD) δ 7.47 – 7.07 (m, 8H), 4.45 (q, J=6 Hz, 1H), 4.24 (s, 2H), 2.16 (s, 6H), 1.63 (d, J=6 Hz, 3H) ppm. ^{13}C NMR (75 MHz, $CDCl_3$) δ 170.60, 157.85, 137.95, 136.83, 129.98, 129.30, 128.08, 127.28, 126.21, 52.12, 47.09, 20.68, 18.17 ppm. *m/z* (MALDI-TOF MS): calcd for $C_{19}H_{23}N_3O_2$ $[M + H]^+$: 326.1790, found: 326.1793.

***N*-(3-chlorobenzyl)-*N′*-(1-phenylethyl)urea 16 (Table 3, entry 7):**

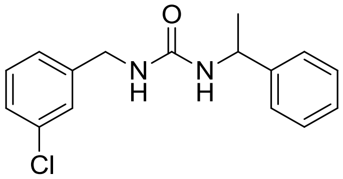

Yield 86 mg (94%); a withe solid. ^1H NMR (300 MHz, $CDCl_3$) δ 7.32 – 6.97 (m, 9H), 4.76 (q, J=6 Hz, 1H), 3.76 (s, 2H), 1.41 (d, J=6 Hz, 3H) ppm. ^{13}C NMR (75 MHz, $CDCl_3$) δ 158.97, 140.16, 134.72, 134.70, 130.20, 129.13, 127.88, 127.26, 125.94, 125.38, 64.00, 43.98, 23.59 ppm. *m/z* (MALDI-TOF MS): calcd for $C_{16}H_{17}ClN_2O$ $[M + H]^+$: 289.1029, found: 289.1033.

***N,N′*-bis-benzylurea 27 (Table 4, entry 1)** [1]:

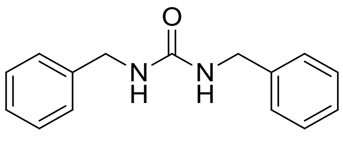

Yield 74 mg (98%); a white solid. ^{1}H NMR (300 MHz, $CDCl_3$) δ 7.36 – 7.23 (m, 10H), 4.38 (s, 3H) ppm. ^{13}C NMR (75 MHz, $CDCl_3$) δ 158.78, 139.06, 128.95, 127.67, 127.65, 44.82 ppm. *m/z* (MALDI-TOF MS): calcd for $C_{15}H_{16}N_2O$ $[M + H]^+$: 241.1262, found: 241.1259.

***N*-benzylmorpholine-4 carboxamide 28 (Table 4, entry 2)** [3]**:**

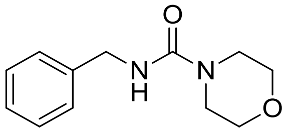

Yield 68 mg (98%); a yellowish solid. ^{1}H NMR (300 MHz, $CDCl_3$) δ 7.28 – 7.20 (m, 5H), 4.38 (s, 2H), 3.63 (t, *J*=6 Hz, 4H), 3.30 (t, *J*=6 Hz, 4H) ppm. ^{13}C NMR (75 MHz, $CDCl_3$) δ 157.75, 139.27, 129.04, 120.16, 127.83, 66.68, 45.38, 44.60 ppm. *m/z* (MALDI-TOF MS): calcd for $C_{12}H_{16}N_2O_2$ $[M + H]^+$: 221.1212, found: 221.1219.

N-benzyl-*N′*-phenylurea **29 (Table 4, entry 3)** [4]**:**

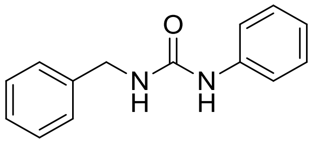

Yield 69 mg (98%); a yellowish solid. ^{1}H NMR (300 MHz, $CDCl_3$) δ 7.35 – 7.13 (m, 10H), 4.44 (s, 2H) ppm. ^{13}C NMR (75 MHz, $CDCl_3$) δ 156.72, 129.72, 129.20, 129.04, 127.81, 127.76, 127.62, 124.74, 121.97, 44.64 ppm. *m/z* (MALDI-TOF MS): calcd for $C_{14}H_{14}N_2O$ $[M + H]^+$: 227.1106, found: 227.1100.

***N*-(1-phenylethyl)morpholine-4-carboxamide 30 (Table 4, entry 5)** [3]:

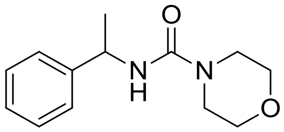

Yield 69 mg (94%); a yellowish solid. ^{1}H NMR (300 MHz, $CDCl_3$) δ 7.30 – 7.17 (m, 5H), 4.95 (q, *J*=6 Hz, 1H), 3.62 (t, *J*=6 Hz, 4H), 3.27 (t, *J*=6 Hz, 4H), 1.43 (d, *J*=6 Hz, 3H) ppm. ^{13}C NMR (75 MHz, $CDCl_3$) δ 157.14, 144.36, 144.36, 128.99, 127.59, 126.45, 66.71, 50.51, 44.45, 22.84 ppm. *m/z* (MALDI-TOF MS): calcd for $C_{13}H_{18}N_2O_2$ $[M + H]^+$: 235.1368, found: 235.1372.

***N*-(1-phenylethyl)-*N′*-phenylurea 31 (Table 4, entry 6):**

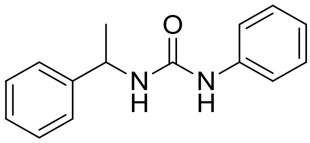

Yield 75 mg (98%); a yellowish solid. ^{1}H NMR (300 MHz, $CDCl_3$) δ 7.37 – 7.06 (m, 10H), 4.95 (q, *J*=6 Hz, 1H), 1.48 (d, *J*=6 Hz, 3H) ppm. ^{13}C NMR (75 MHz, $CDCl_3$) δ 155.86, 144.06, 138.61,

129.51, 129.06, 127.66, 126.23, 124.07, 121.12, 50.41, 23.28 ppm. *m/z* (MALDI-TOF MS): calcd for $C_{15}H_{16}N_2O$ $[M + H]^+$: 241.1262, found: 241.1256.

***N*-(1-phenylethyl)-3,4-dihydroisoquinoline-2-carboxamide 32 (Table 4, entry 7):**

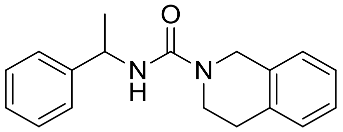

Yield 87 mg (98%); a withe solid. ^{1}H NMR (300 MHz, $CDCl_3$) δ 7.28 – 6.99 (m, 9H), 4.99 (q, *J*=6 Hz, 1H), 4.44 (s, 2H), 3.52 (t, *J*=6 Hz, 2H), 2.76 (t, *J*=6 Hz, 2H), 1.43 (d, *J*=6 Hz, 3H) ppm. ^{13}C NMR (75 MHz, $CDCl_3$) δ 157.03, 144.71, 135.35, 133.59, 128.86, 128.64, 127.38, 126.92, 126.64, 126.60, 126.44, 50.38, 45.77, 41.46, 29.31, 22.92 ppm. *m/z* (MALDI-TOF MS): calcd for $C_{18}H_{20}N_2O$ $[M + H]^+$: 281.1575, found: 281.1578.

***N*-(1-phenylethyl)-*N'*-(2-chlorobenzyl)urea 33 (Table 4, entry 8):**

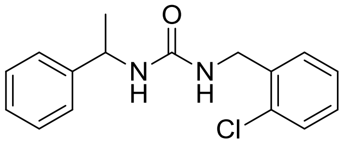

Yield 89 mg (98%); a yellowish solid. ^{1}H NMR (300 MHz, $CDCl_3$) δ 7.27 – 7.11 (m, 9H), 4.73 (q, *J*=6 Hz, 1H), 4.30 (s, 2H), 1.37 (d, *J*=6 Hz, 3H) ppm. ^{13}C NMR (75 MHz, $CDCl_3$) δ 158.20, 129.59, 129.48, 129.02, 128.76, 127.58, 127.26, 126.12, 50.76, 42.36, 23.70 ppm. *m/z* (MALDI-TOF MS): calcd for $C_{16}H_{17}ClN_2O$ $[M + H]^+$: 289.1029, found: 289.1031.

***N*-Octyl-*N'*-benzylurea 34 (Table 4, entry 9):**

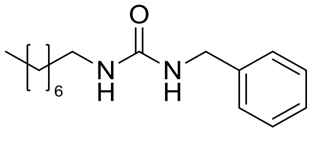

Yield 74 mg (89%); a yellowish oil. ^{1}H NMR (300 MHz, $CDCl_3$) δ 7.34 – 7.26 (m, 5H), 4.38 (s, 2H), 3.15 (t, *J*=6 Hz, 2H), 1.47 (m, 2H), 1.25 (o.s., 10H), 0.82 (t, *J*=6 Hz, 3H) ppm. ^{13}C NMR (75 MHz, $CDCl_3$) δ 158.92, 139.46, 128.92, 127.66, 127.59, 44.77, 40.99, 32.13, 30.44, 29.56, 27.19, 22.98, 14.43 ppm. *m/z* (MALDI-TOF MS): calcd for $C_{16}H_{26}N_2O$ $[M + H]^+$: 263.2045, found: 263.2041.

***N*-Octyl-morpholine-4-carboxamide 35 (Table 4, entry 10):**

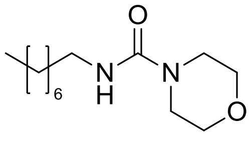

Yield 68 mg (89%); a yellowish oil. ^{1}H NMR (300 MHz, $CDCl_3$) δ 3.70 (t, J=6 Hz, 4H), 3.33 (t, *J*=6 Hz, 4H), 3.22 (t, *J*=6 Hz, 2H), 1.50 (m, 2H), 1.35 – 1.17 (o.s., 10H), 0.87 (t, J=6 Hz, 3H) ppm.

^{13}C NMR (75 MHz, $CDCl_3$) δ 158.20, 66.78, 44.27, 41.28, 32.09, 30.49, 29.52, 27.25, 22.93, 14.40 ppm. *m/z* (MALDI-TOF MS): calcd for $C_{13}H_{26}N_2O_2$ $[M + H]^+$: 243.1994, found: 243.1996.

***N*-Octyl-*N'*-phenylurea 36 (Table 4, entry 11)** [5]**:**

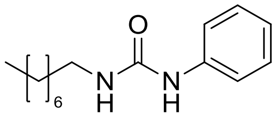

Yield 69 mg (88%); a yellowish oil. ^1H NMR (300 MHz, $CDCl_3$) δ 7.34 – 7.09 (m, 5H), 3.23 (t, *J*=6 Hz, 2H), 1.49 (m, 2H), 1.26 (o.s., 10H), 0.87 (t, *J*=6 Hz, 3H) ppm. ^{13}C NMR (75 MHz, $CDCl_3$) δ 156.84, 138.91, 129.50, 123.97, 121.28, 40.77, 32.14, 30.41, 29.57, 27.24, 22.98, 14.44 ppm. *m/z* (MALDI-TOF MS): calcd for $C_{15}H_{24}N_2O$ $[M + H]^+$: 249.1888, found: 249.1893.

***N*-(*sec*-butyl)-*N'*-(1-phenylethyl)urea 37 (Table 4, entry 12):**

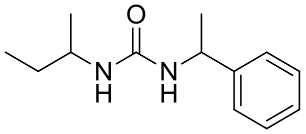

Yield 59 mg (85%); a yellowish solid. ^1H NMR (300 MHz, CD_3OD) δ 7.32 – 7.21 (m, 5H), 4.84 (q, *J*=6 Hz, 1H), 3.61 (m, *J*=6 Hz, 1H), 1.43 (m, 5H), 1.09 (d, *J*=6 Hz, 3H), 0.87 (t, *J*=6 Hz, 3H) ppm. ^{13}C NMR (75 MHz, $CDCl_3$) δ 158.72, 143.87, 128.98, 127.56, 126.02, 51.31, 48.90, 29.97, 20.92, 20.84, 10.58 ppm. *m/z* (MALDI-TOF MS): calcd for $C_{13}H_{20}N_2O$ $[M + H]^+$: 221.1575, found: 221.1577.

***N*-allyl-*N'*-(1-phenylethyl)urea 38 (Table 4, entry 13):**

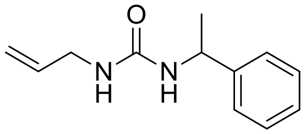

Yield 54 mg (83%); a yellowish solid. ^1H NMR (300 MHz, $CDCl_3$) δ 7.31 – 7.20 (m, 5H), 5.70 (m, 1H), 5.00 (m, 2H), 4.73 (q, *J*=6 Hz, 1H), 4.08 (d, *J*=9 Hz, 2H), 1.38 (d, *J*=6 Hz, 3H) ppm. ^{13}C NMR (75 MHz, $CDCl_3$) δ 158.30, 144.44, 135.47, 120.02, 127.57, 126.17, 115.75, 50.58, 43.10, 23.70 ppm. *m/z* (MALDI-TOF MS): calcd for $C_{12}H_{16}N_2O$ $[M + H]^+$: 205.1262, found: 205.1266.

2. ^{1}H and ^{13}C NMR spectra

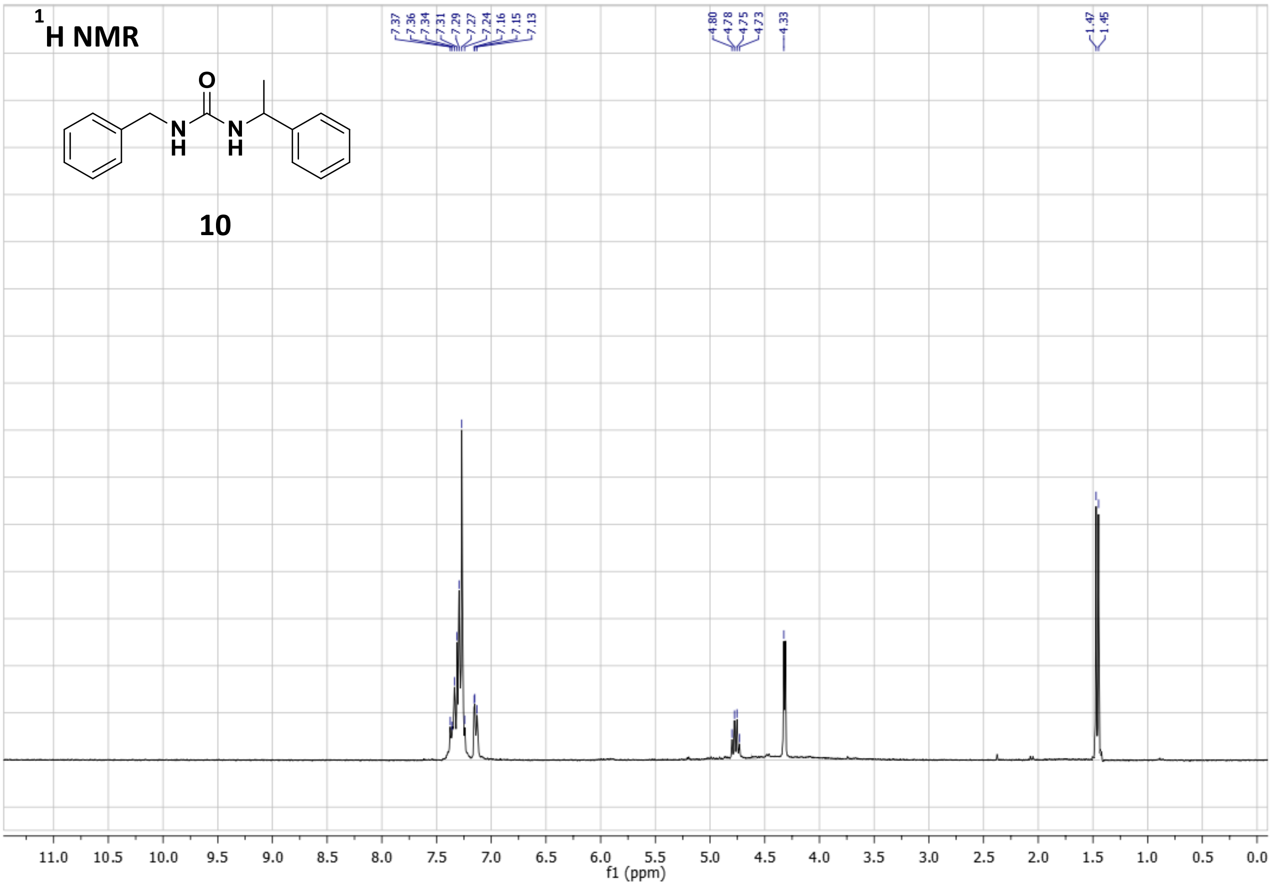

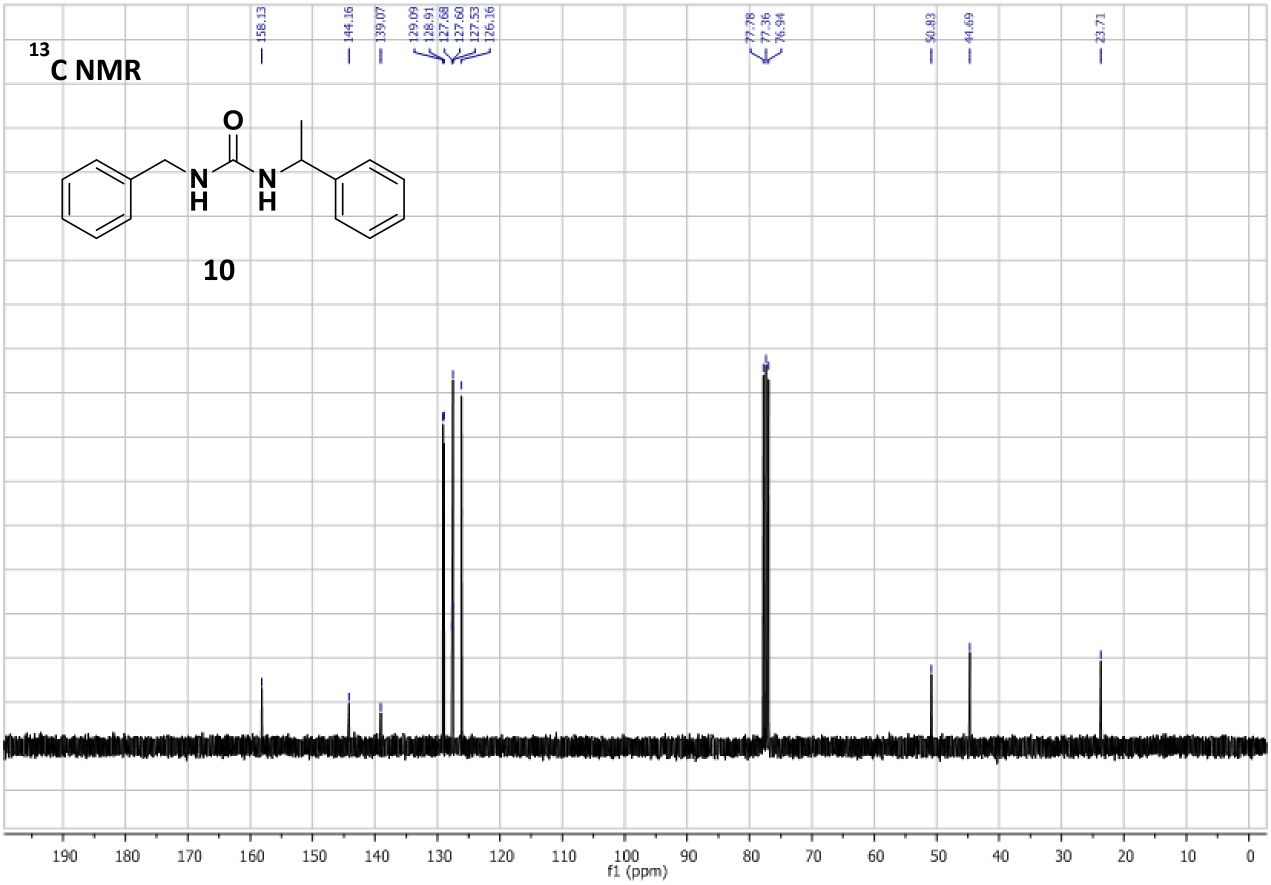

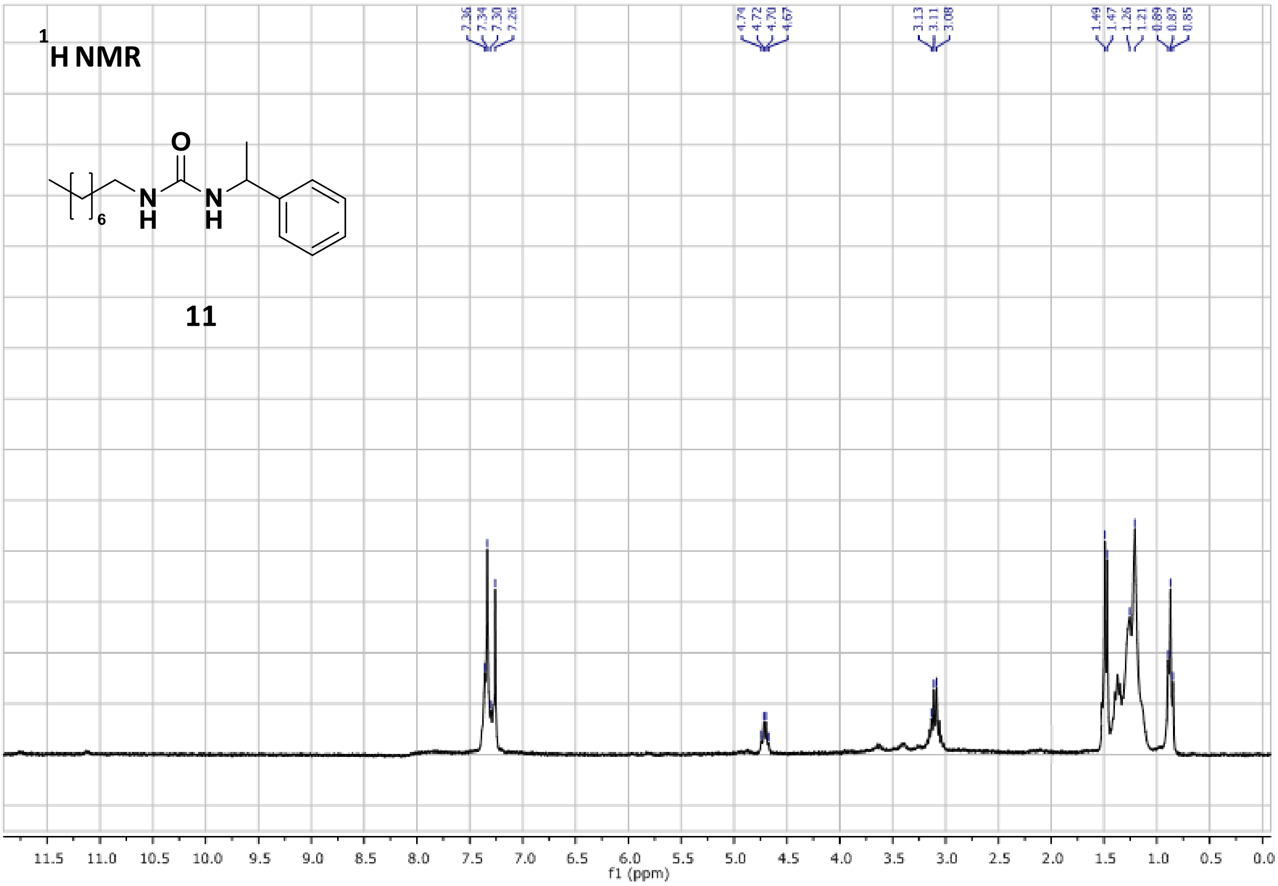
1H NMR
O
N
H
N
H
6
11
7.36 7.34 7.30 7.26
4.74 4.72 4.70 4.67
3.13 3.11 3.08
1.49 1.47 1.26 1.21 0.89 0.87 0.85
f1 (ppm)

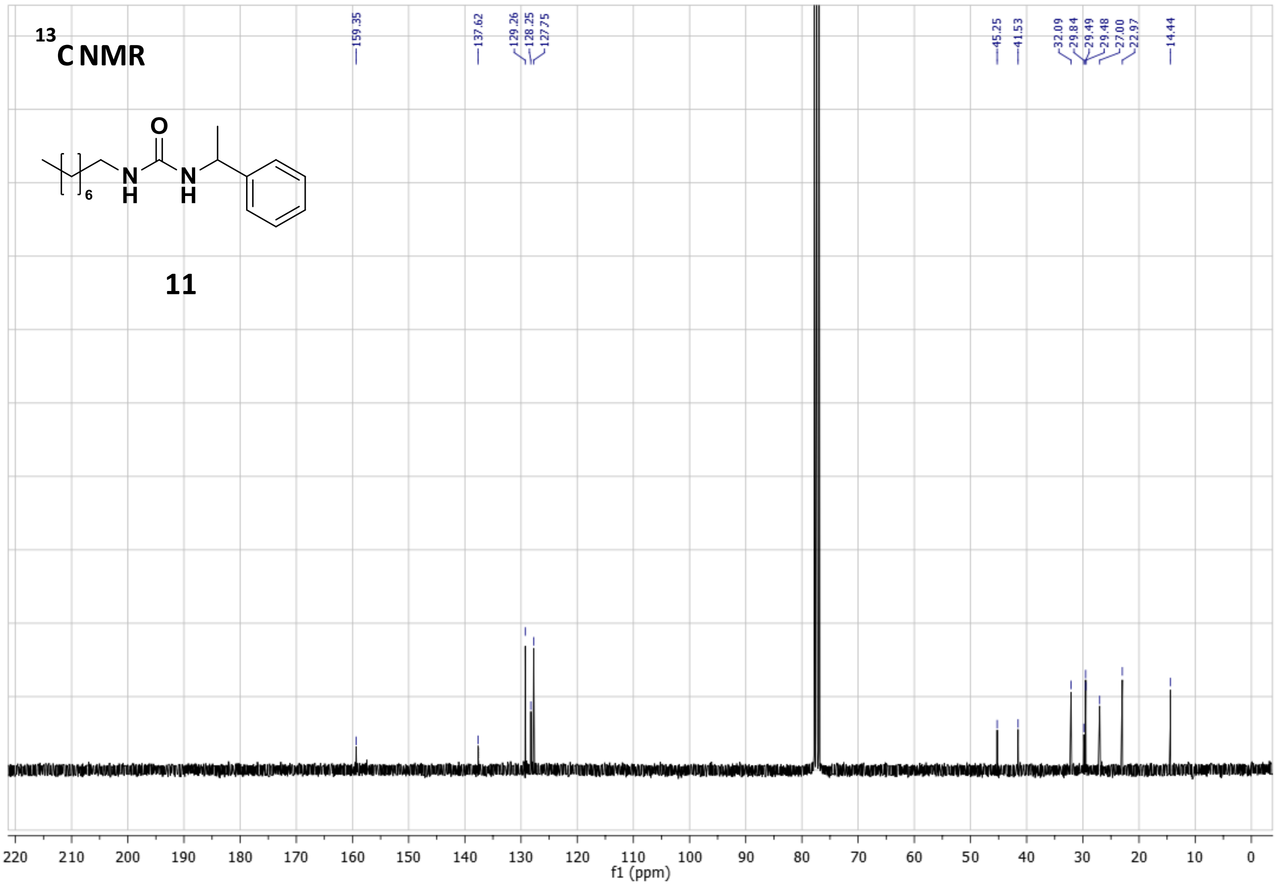
13C NMR
O
N
H
N
H
6
11
159.35
137.62
129.26 128.25 127.75
45.25
41.53
32.09 29.84 29.49 29.48 27.00 22.97
14.44
f1 (ppm)

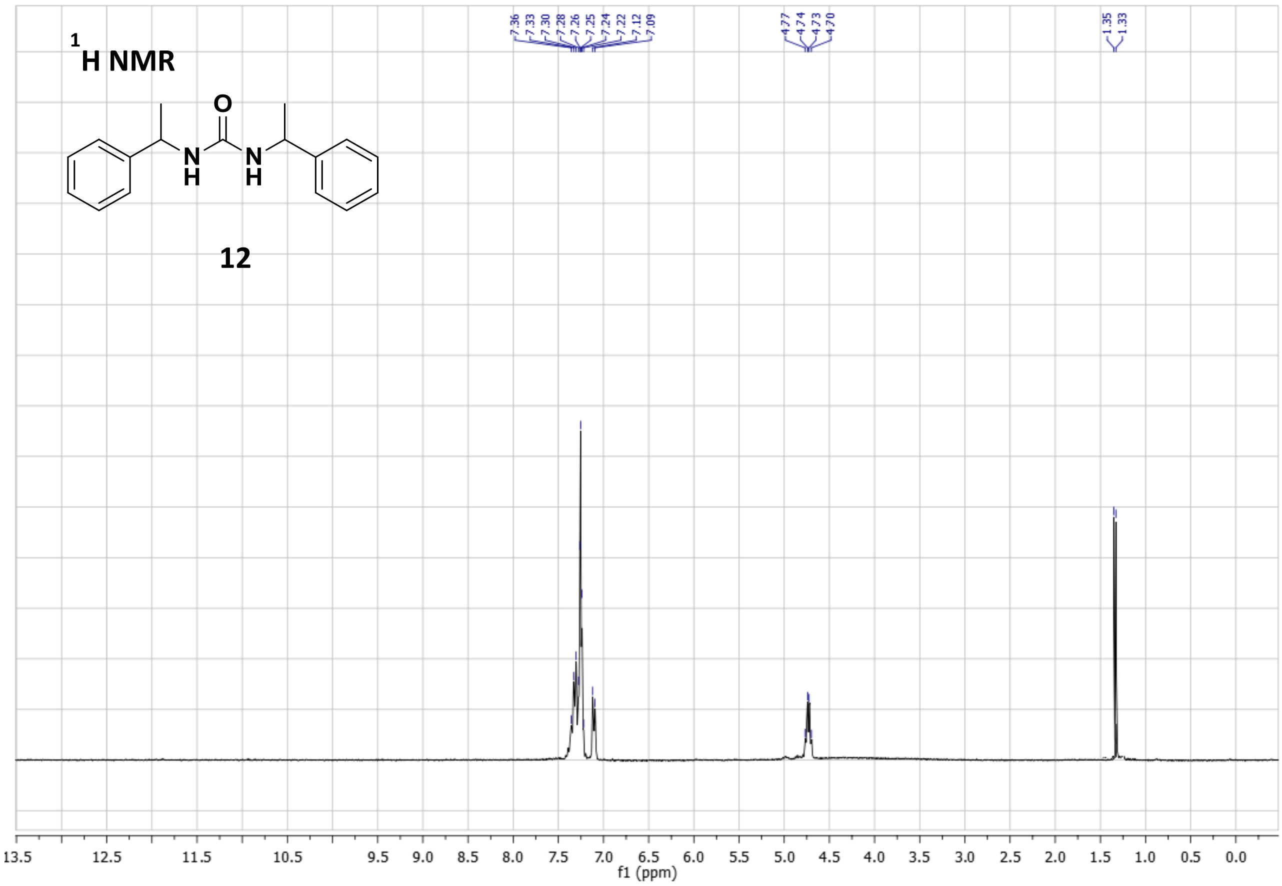
1H NMR
O
N
H
N
H
12
7.36
7.33
7.30
7.28
7.26
7.25
7.24
7.22
7.12
7.09
4.77
4.74
4.73
4.70
1.35
1.33
13.5
12.5
11.5
10.5
9.5
9.0
8.5
8.0
7.5
7.0
6.5
6.0
5.5
5.0
4.5
4.0
3.5
3.0
2.5
2.0
1.5
1.0
0.5
0.0
f1 (ppm)

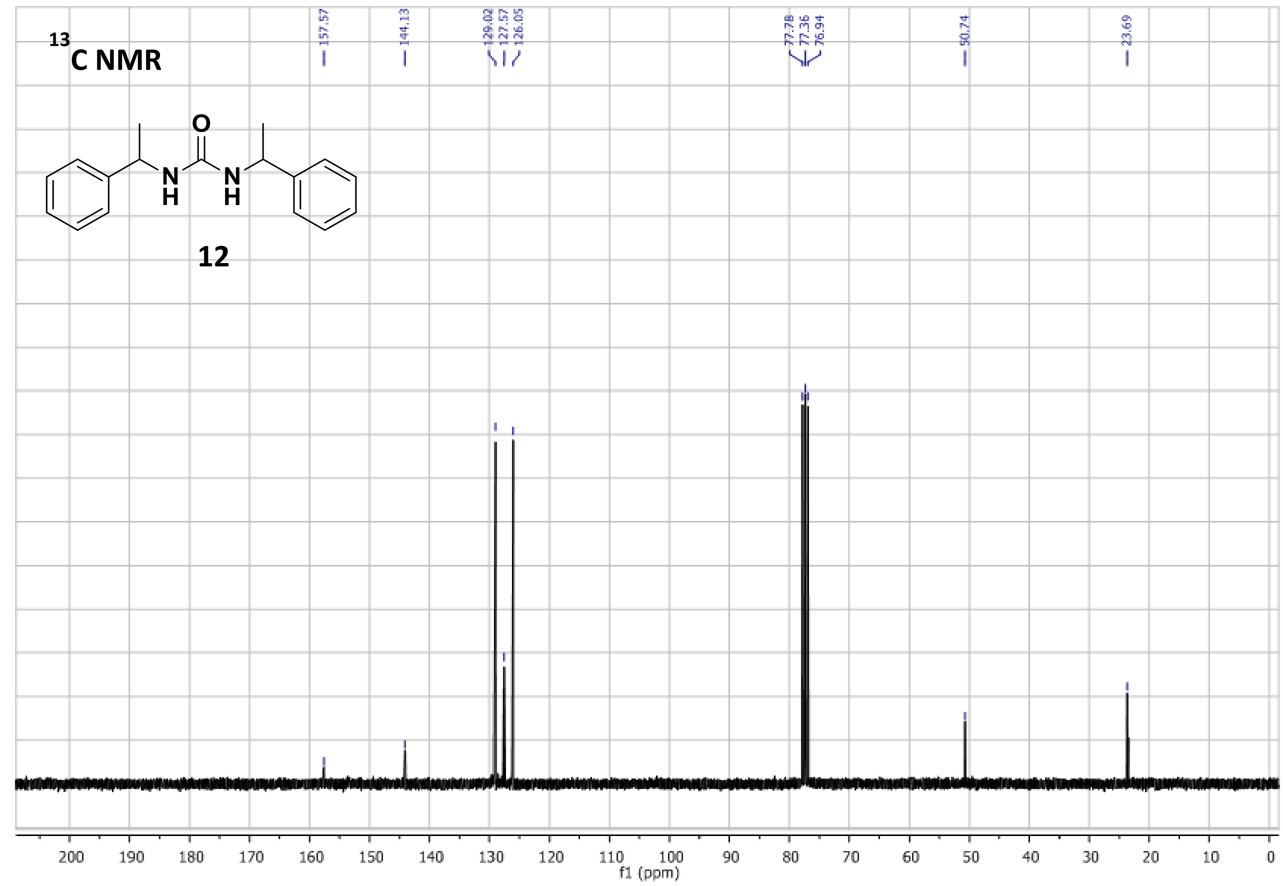
13C NMR
O
N
H
N
H
12
157.57
144.13
129.02
127.57
126.05
77.78
77.36
76.94
50.74
23.69
200
190
180
170
160
150
140
130
120
110
100
90
80
70
60
50
40
30
20
10
0
f1 (ppm)

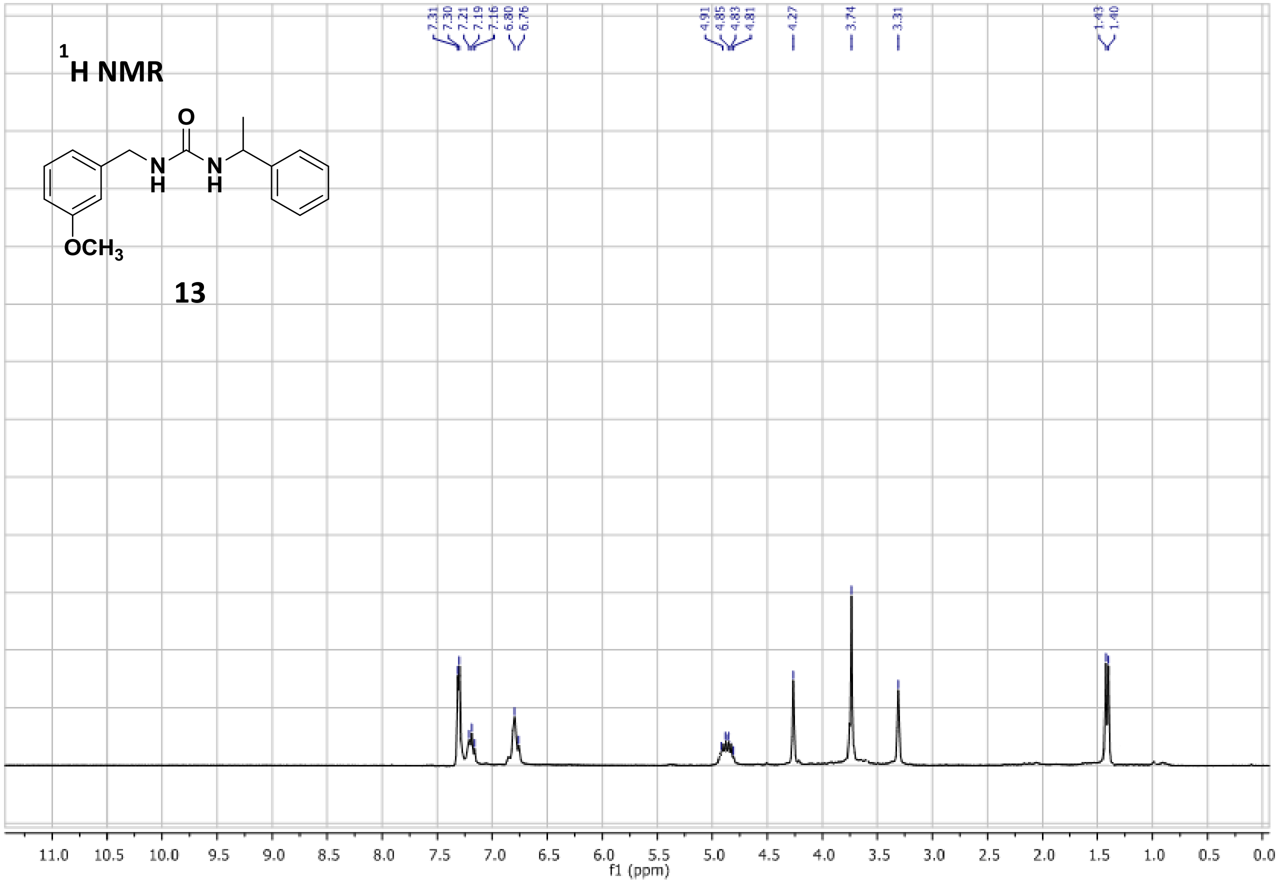

1H NMR
O
N
H
N
H
OCH3
13
7.31
7.30
7.21
7.19
7.16
6.80
6.76
4.91
4.85
4.83
4.81
4.27
3.74
3.31
1.43
1.40
11.0
10.5
10.0
9.5
9.0
8.5
8.0
7.5
7.0
6.5
6.0
5.5
5.0
4.5
4.0
3.5
3.0
2.5
2.0
1.5
1.0
0.5
0.0
f1 (ppm)

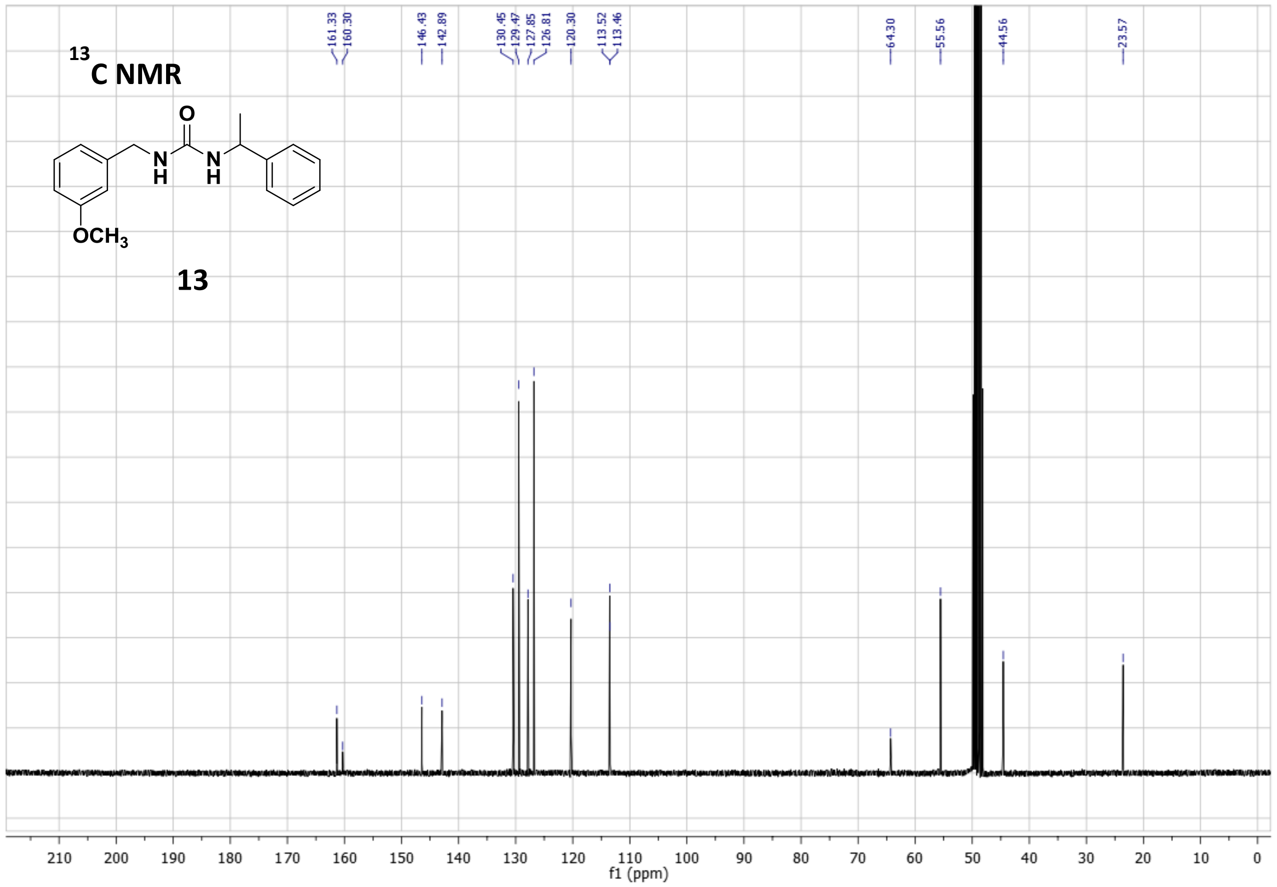

13C NMR
O
N
H
N
H
OCH3
13
161.33
160.30
146.43
142.89
130.45
129.47
127.85
126.81
120.30
113.52
113.46
64.30
55.56
44.56
23.57
210
200
190
180
170
160
150
140
130
120
110
100
90
80
70
60
50
40
30
20
10
0
f1 (ppm)

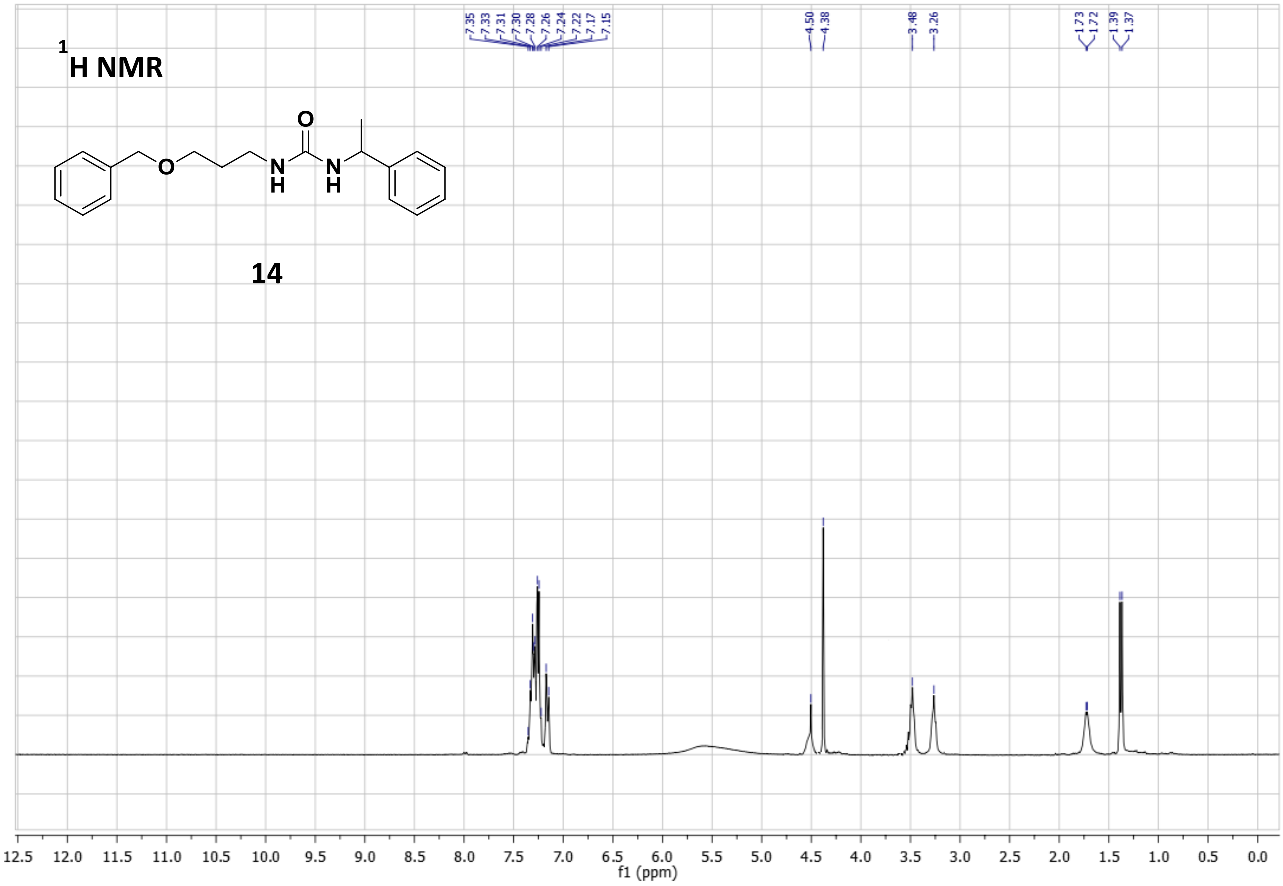

1H NMR
O
O
N
H
N
H
14
7.35 7.33 7.31 7.30 7.28 7.26 7.24 7.22 7.17 7.15
4.50 4.38
3.48 3.26
1.73 1.72 1.39 1.37
12.5 12.0 11.5 11.0 10.5 10.0 9.5 9.0 8.5 8.0 7.5 7.0 6.5 6.0 5.5 5.0 4.5 4.0 3.5 3.0 2.5 2.0 1.5 1.0 0.5 0.0
f1 (ppm)

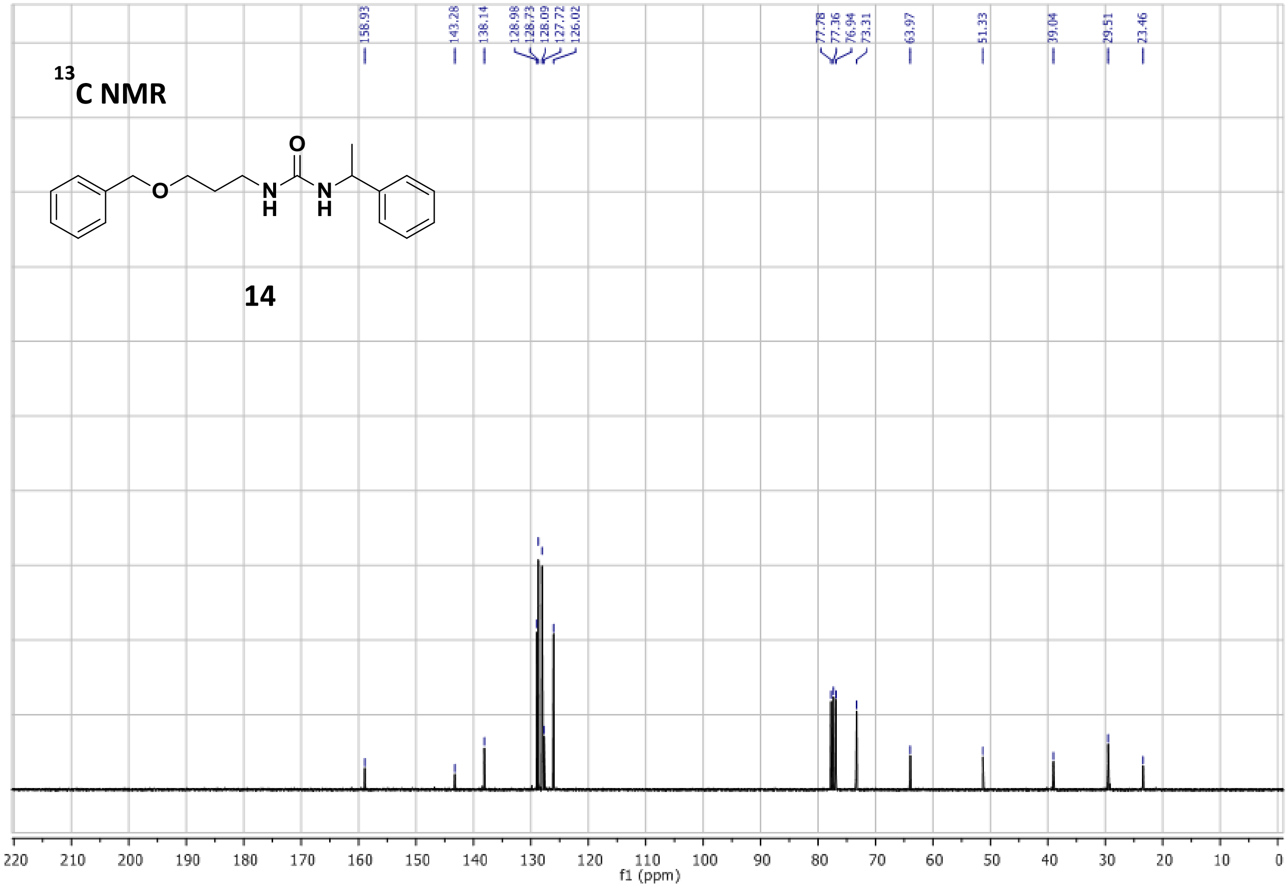

13C NMR
O
O
N
H
N
H
14
158.93
143.28
138.14
128.98 128.73 128.09 127.72 126.02
77.78 77.36 76.94 73.31
63.97
51.33
39.04
29.51
23.46
220 210 200 190 180 170 160 150 140 130 120 110 100 90 80 70 60 50 40 30 20 10 0
f1 (ppm)

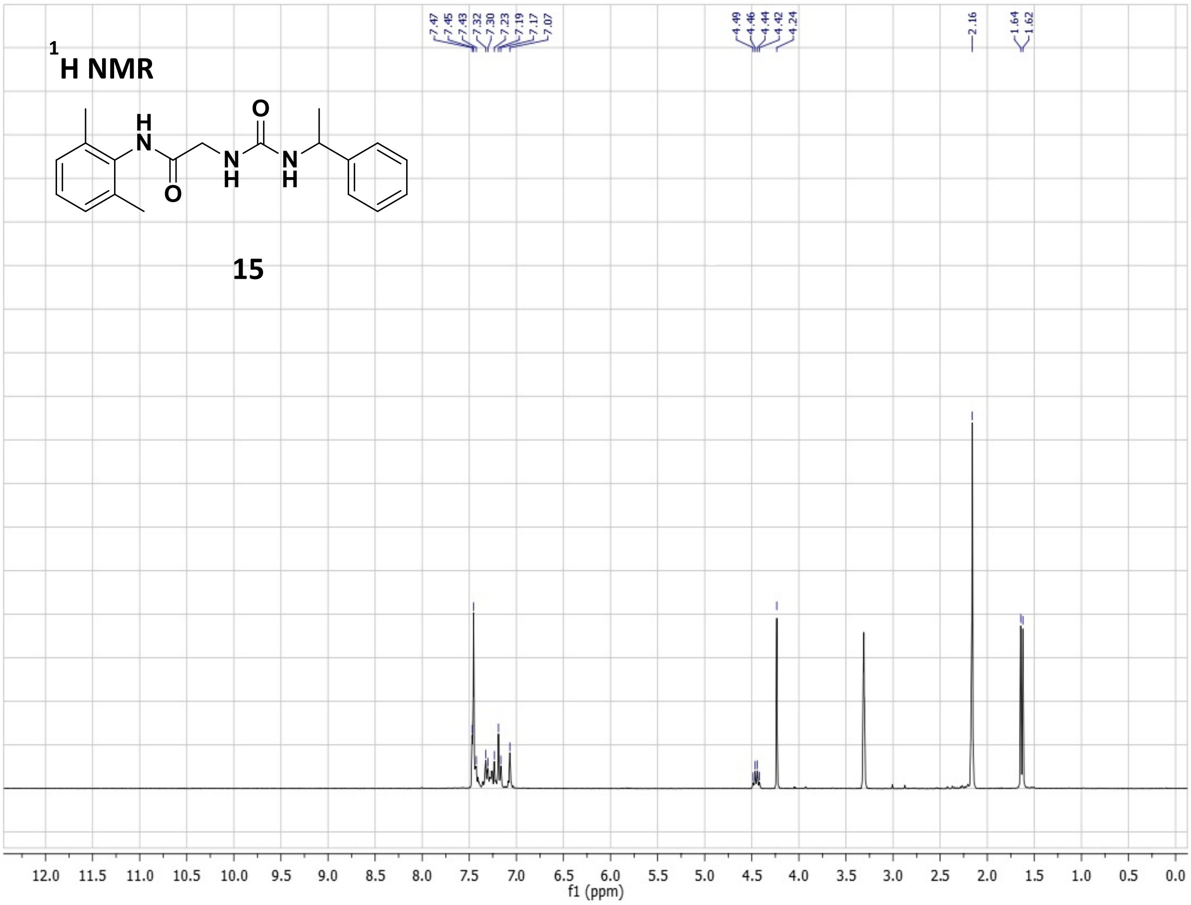

^{1}H NMR
15
7.47 7.45 7.43 7.32 7.30 7.23 7.19 7.17 7.07
4.49 4.46 4.44 4.42 4.24
2.16
1.64 1.62
f1 (ppm)

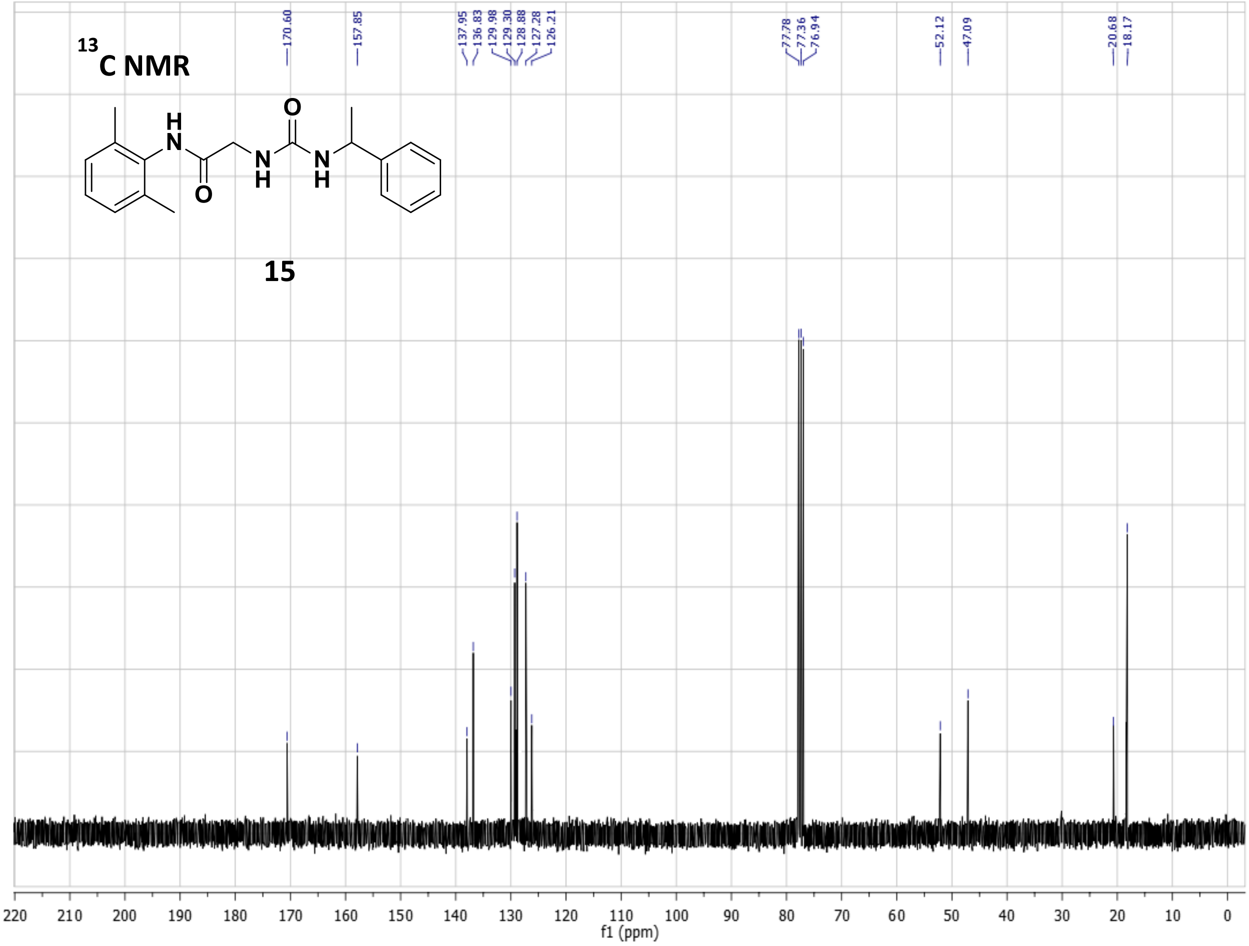

^{13}C NMR
15
170.60
157.85
137.95 136.83 129.98 129.30 128.88 127.28 126.21
77.78 77.36 76.94
52.12
47.09
20.68 18.17
f1 (ppm)

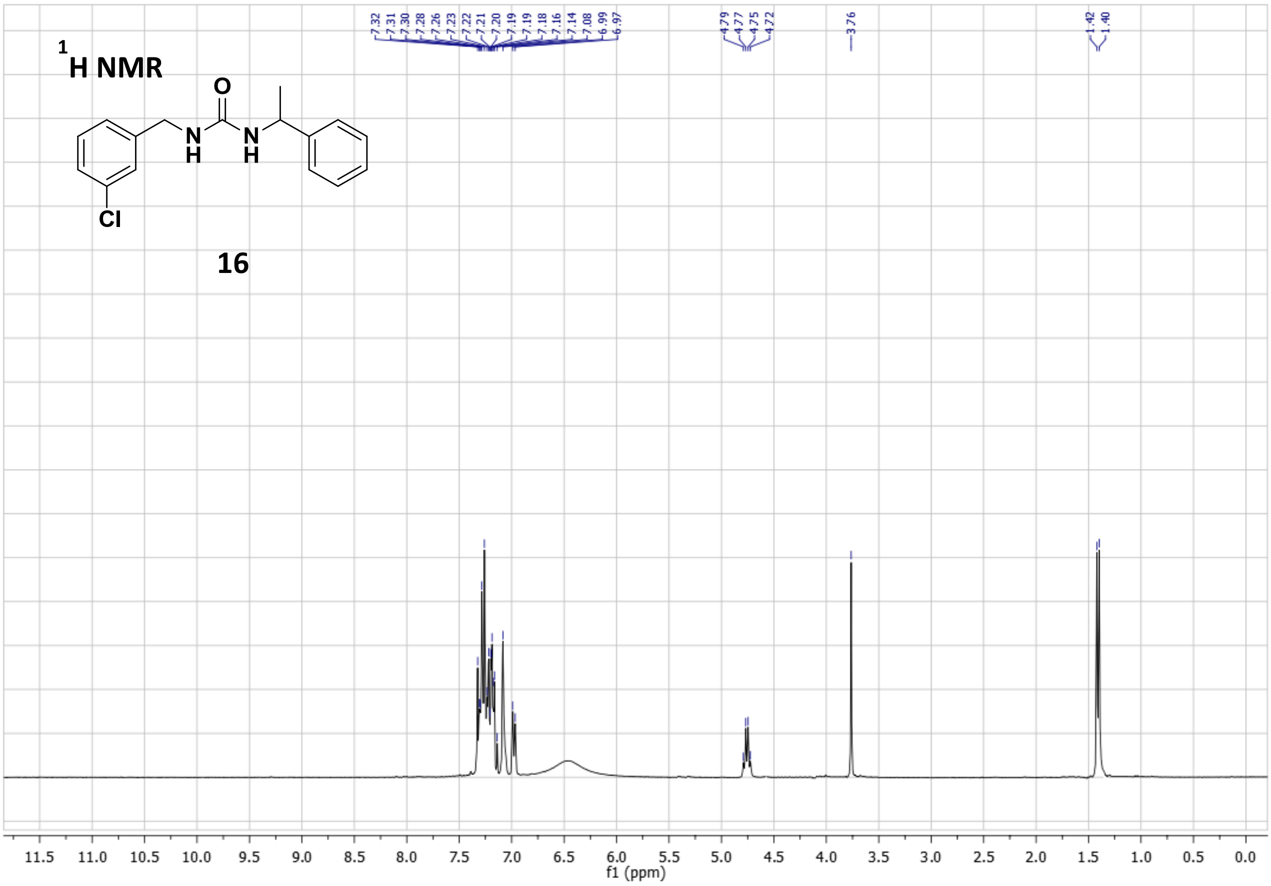
1H NMR
O
N
H
N
H
Cl
16
7.32
7.31
7.30
7.28
7.26
7.23
7.22
7.21
7.20
7.19
7.19
7.18
7.16
7.14
7.08
6.99
6.97
4.79
4.77
4.75
4.72
3.76
1.42
1.40
11.5 11.0 10.5 10.0 9.5 9.0 8.5 8.0 7.5 7.0 6.5 6.0 5.5 5.0 4.5 4.0 3.5 3.0 2.5 2.0 1.5 1.0 0.5 0.0
f1 (ppm)

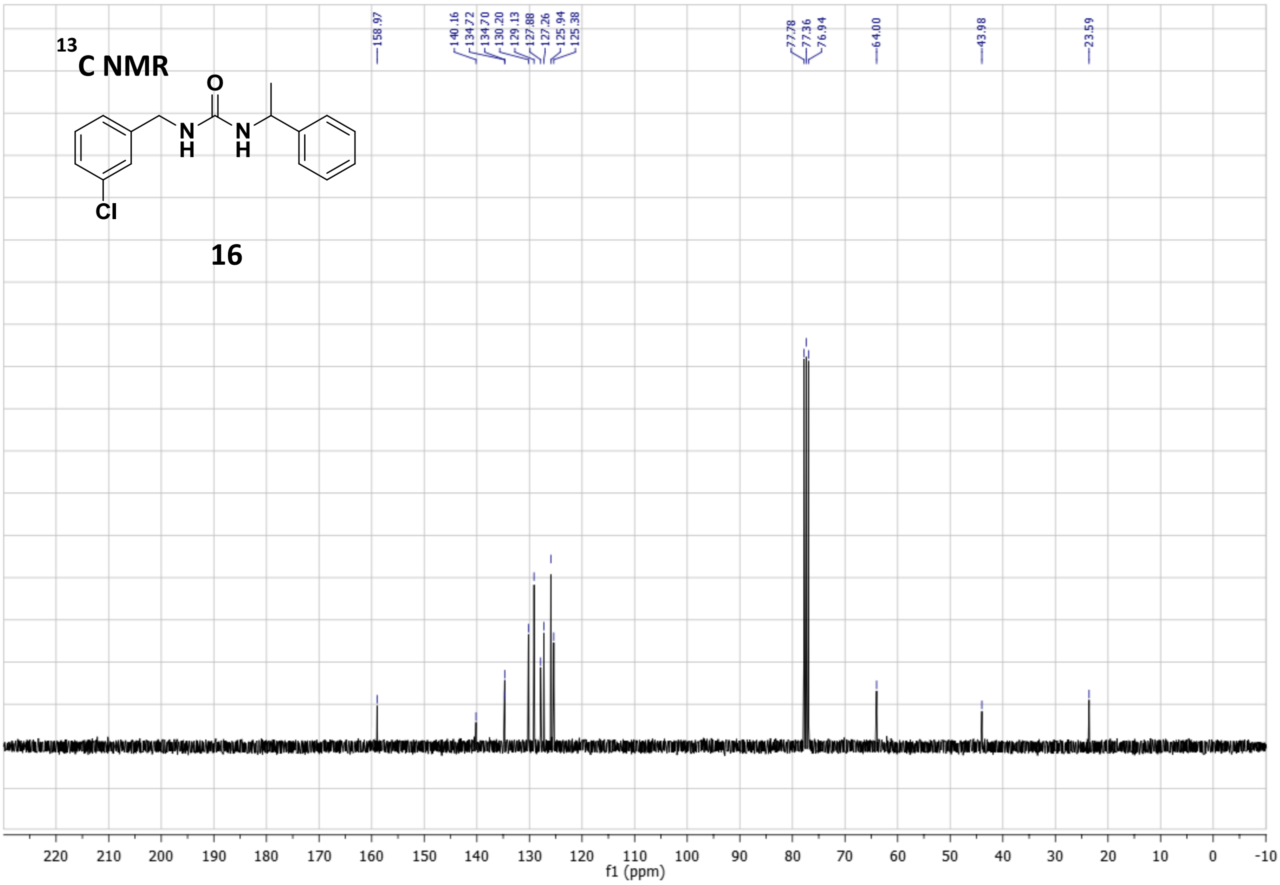
13C NMR
O
N
H
N
H
Cl
16
158.97
140.16
134.72
134.70
130.20
129.13
127.88
127.26
125.94
125.38
77.78
77.36
76.94
64.00
43.98
23.59
220 210 200 190 180 170 160 150 140 130 120 110 100 90 80 70 60 50 40 30 20 10 0 -10
f1 (ppm)

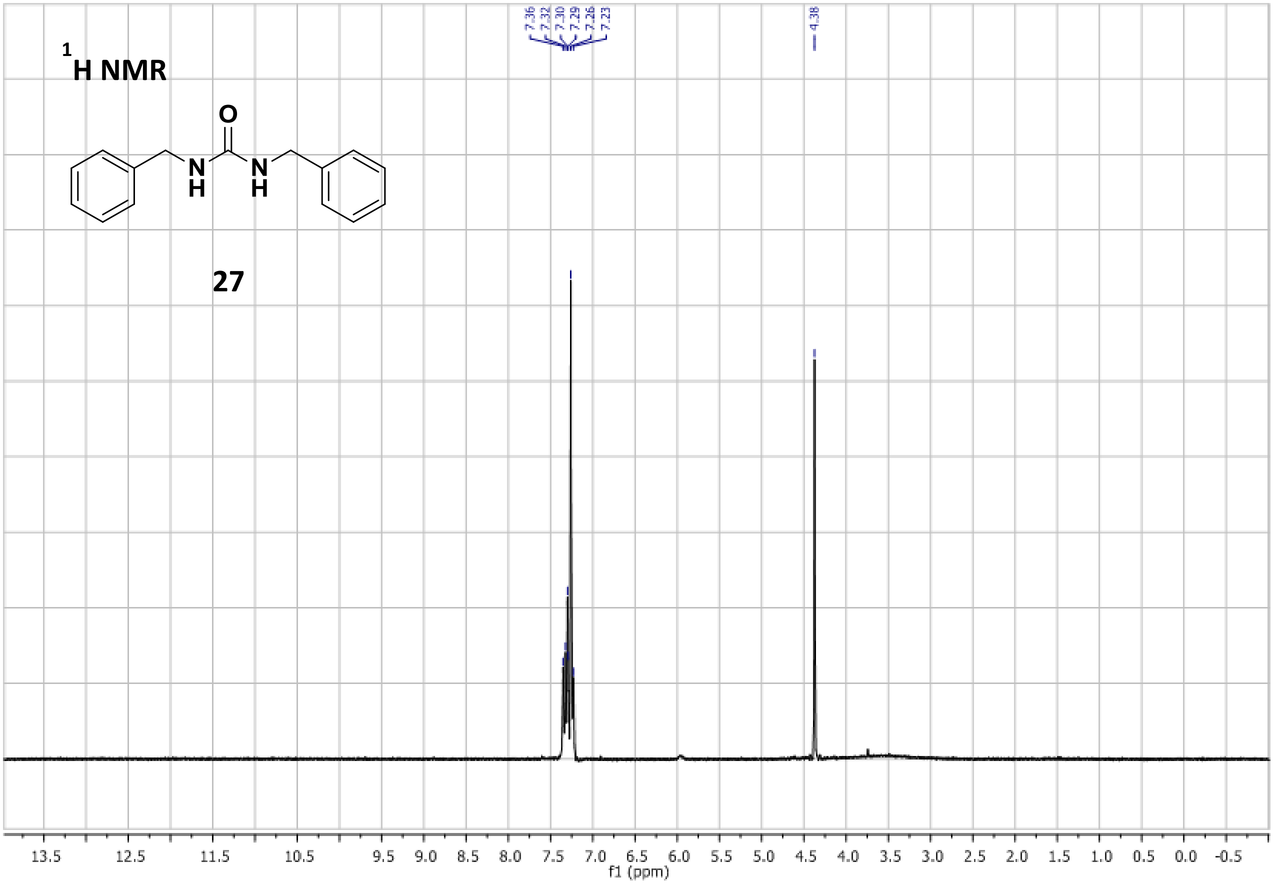
1H NMR
O
N
H
N
H
27
7.36
7.32
7.30
7.29
7.26
7.23
4.38
13.5 12.5 11.5 10.5 9.5 9.0 8.5 8.0 7.5 7.0 6.5 6.0 5.5 5.0 4.5 4.0 3.5 3.0 2.5 2.0 1.5 1.0 0.5 0.0 -0.5
f1 (ppm)

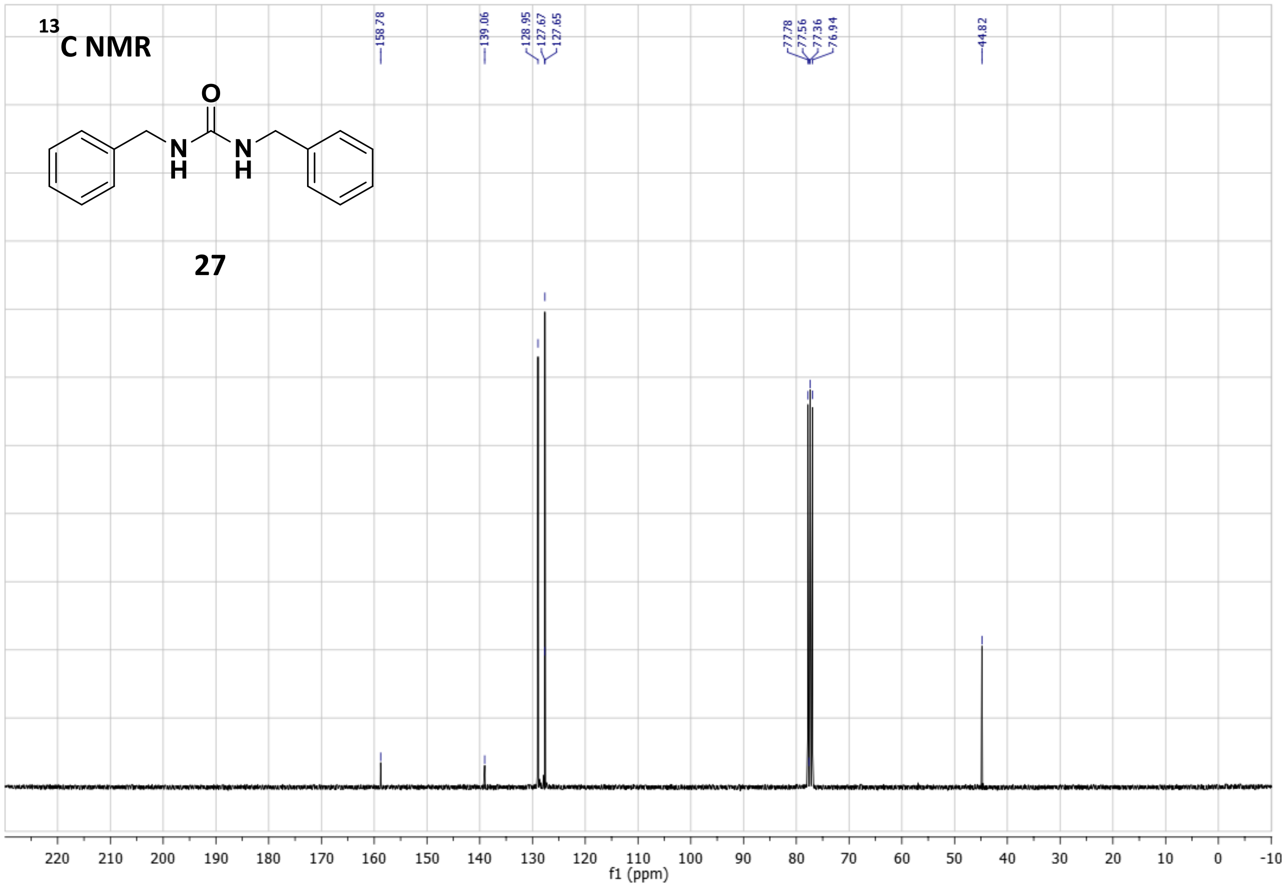
13C NMR
O
N
H
N
H
27
158.78
139.06
128.95
127.67
127.65
77.78
77.56
77.36
76.94
44.82
220 210 200 190 180 170 160 150 140 130 120 110 100 90 80 70 60 50 40 30 20 10 0 -10
f1 (ppm)

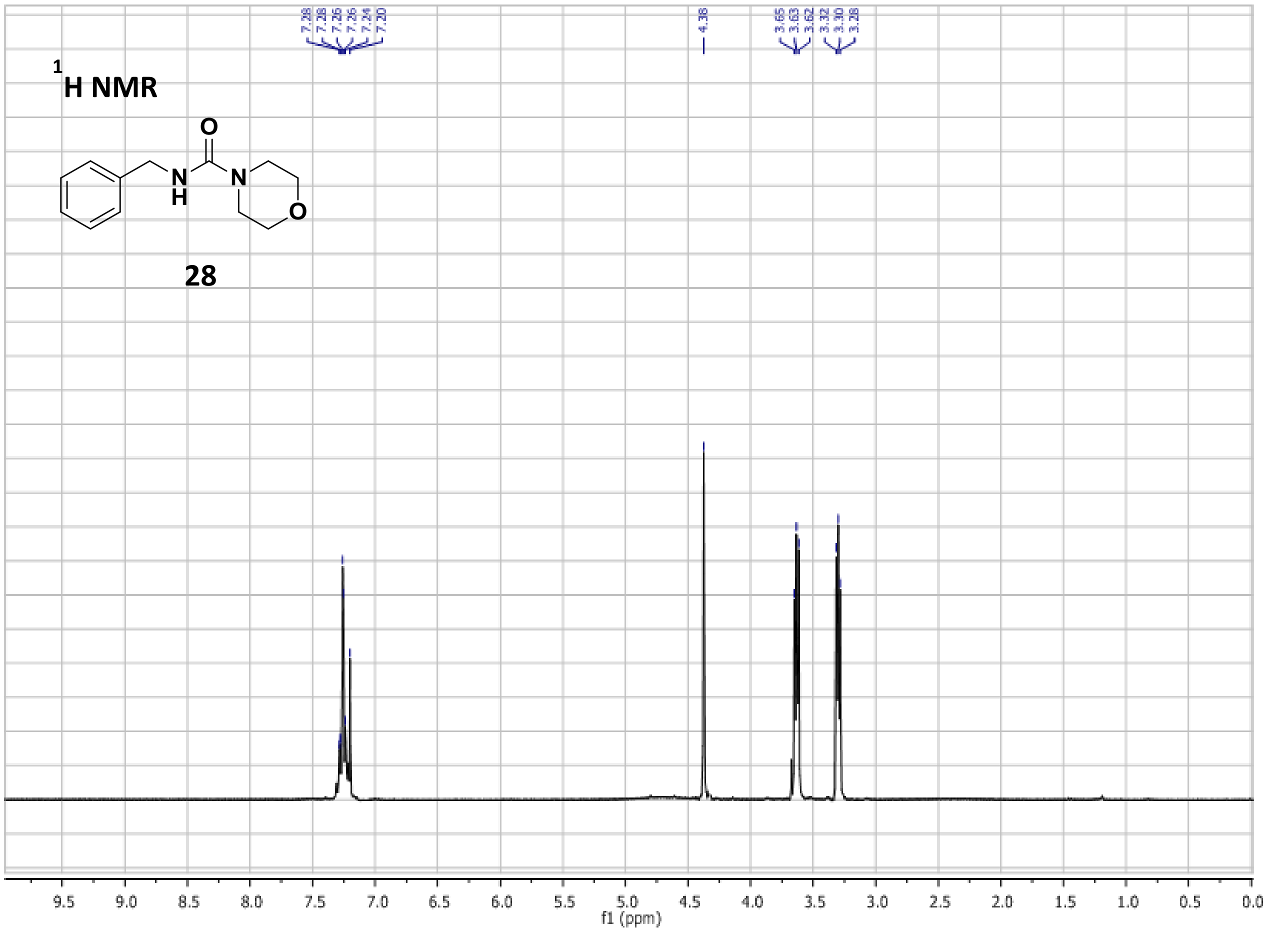
1H NMR
O
N
H
N
O
28
7.28
7.28
7.26
7.26
7.24
7.20
4.38
3.65
3.63
3.62
3.32
3.30
3.28
9.5 9.0 8.5 8.0 7.5 7.0 6.5 6.0 5.5 5.0 4.5 4.0 3.5 3.0 2.5 2.0 1.5 1.0 0.5 0.0
f1 (ppm)

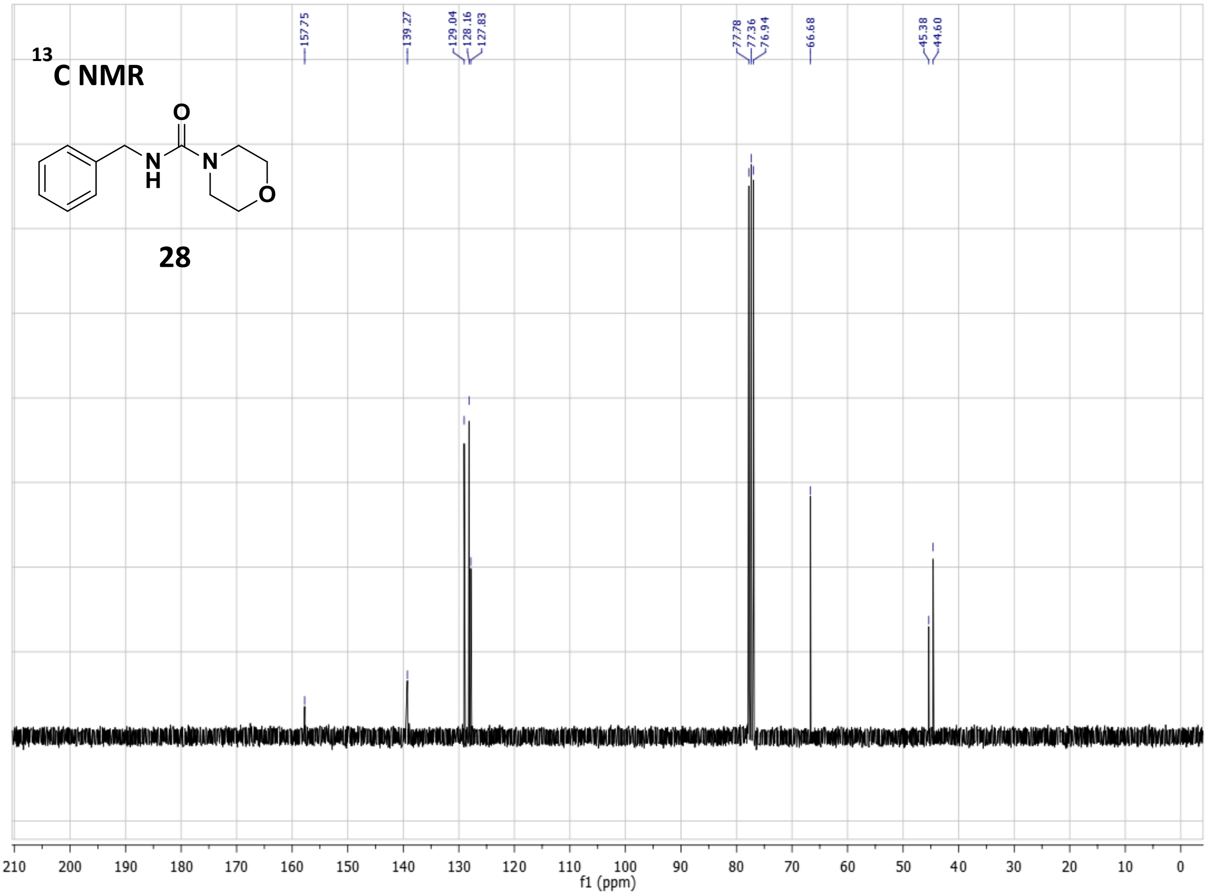
13C NMR
O
N
H
N
O
28
157.75
139.27
129.04
128.16
127.83
77.78
77.36
76.94
66.68
45.38
44.60
210 200 190 180 170 160 150 140 130 120 110 100 90 80 70 60 50 40 30 20 10 0
f1 (ppm)

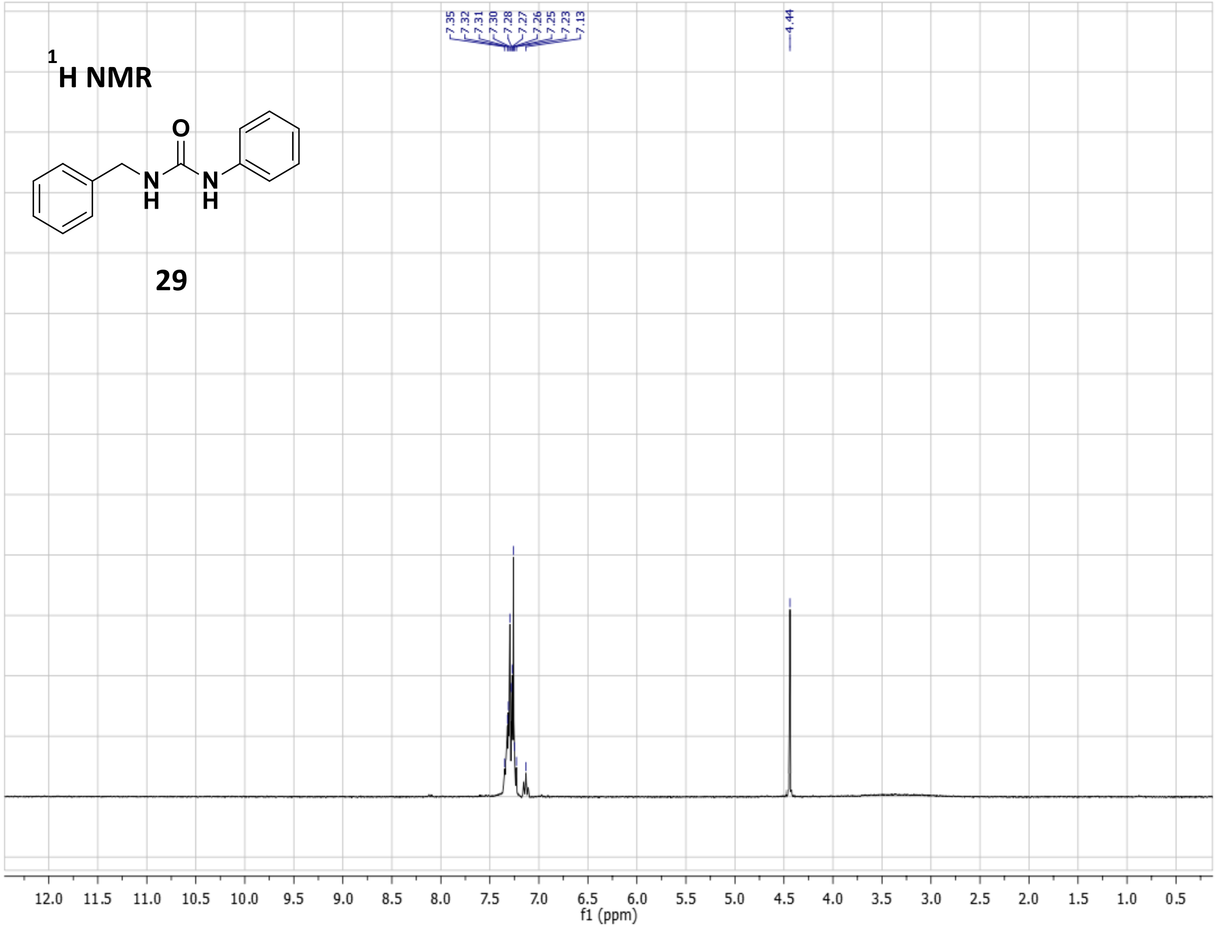

1H NMR
O
N
H
N
H
29
7.35
7.32
7.31
7.30
7.28
7.27
7.26
7.25
7.23
7.13
4.44
12.0 11.5 11.0 10.5 10.0 9.5 9.0 8.5 8.0 7.5 7.0 6.5 6.0 5.5 5.0 4.5 4.0 3.5 3.0 2.5 2.0 1.5 1.0 0.5
f1 (ppm)

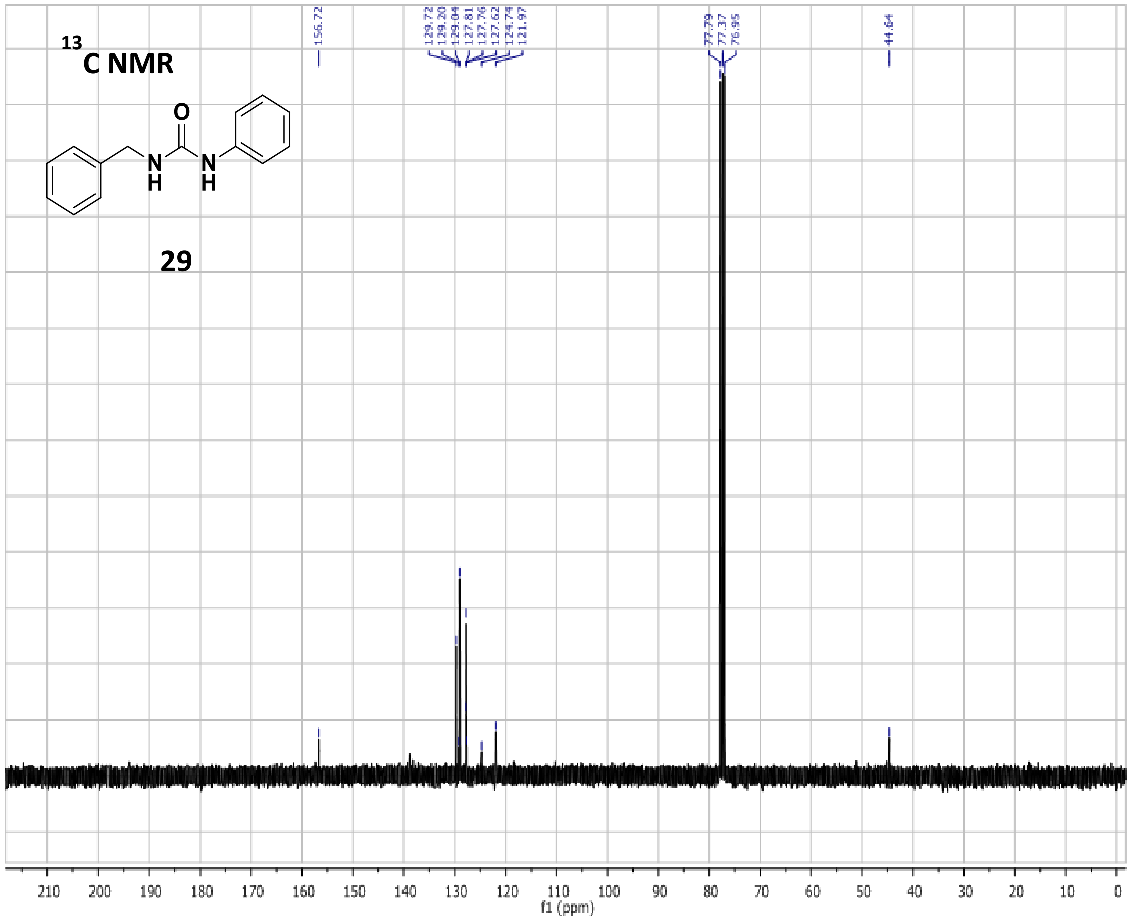

13C NMR
O
N
H
N
H
29
156.72
129.72
129.20
129.04
127.81
127.76
127.62
124.74
121.97
77.79
77.37
76.95
44.64
210 200 190 180 170 160 150 140 130 120 110 100 90 80 70 60 50 40 30 20 10 0
f1 (ppm)

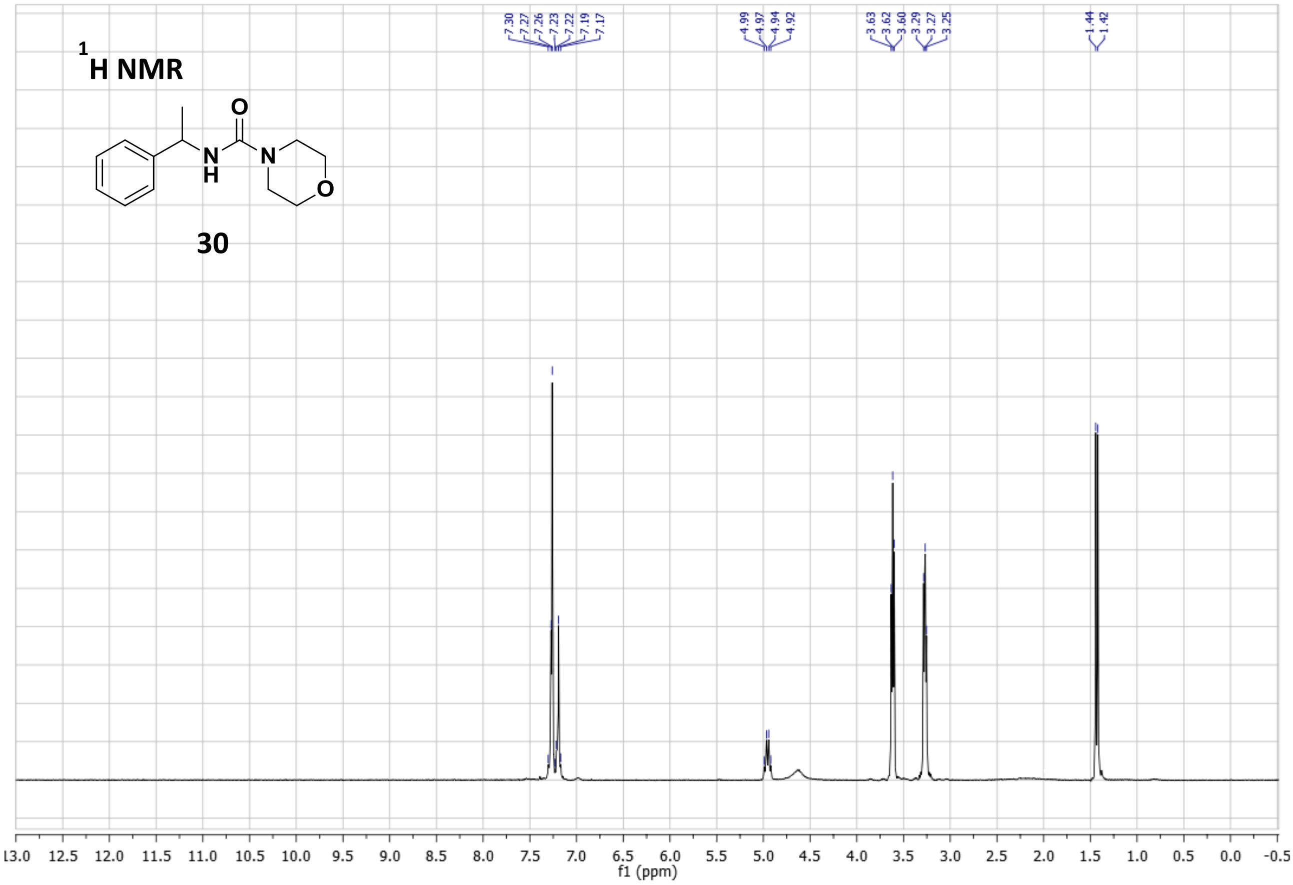

1H NMR
O
N
H
N
O
30
7.30
7.27
7.26
7.23
7.22
7.19
7.17
4.99
4.97
4.94
4.92
3.63
3.62
3.60
3.29
3.27
3.25
1.44
1.42
f1 (ppm)

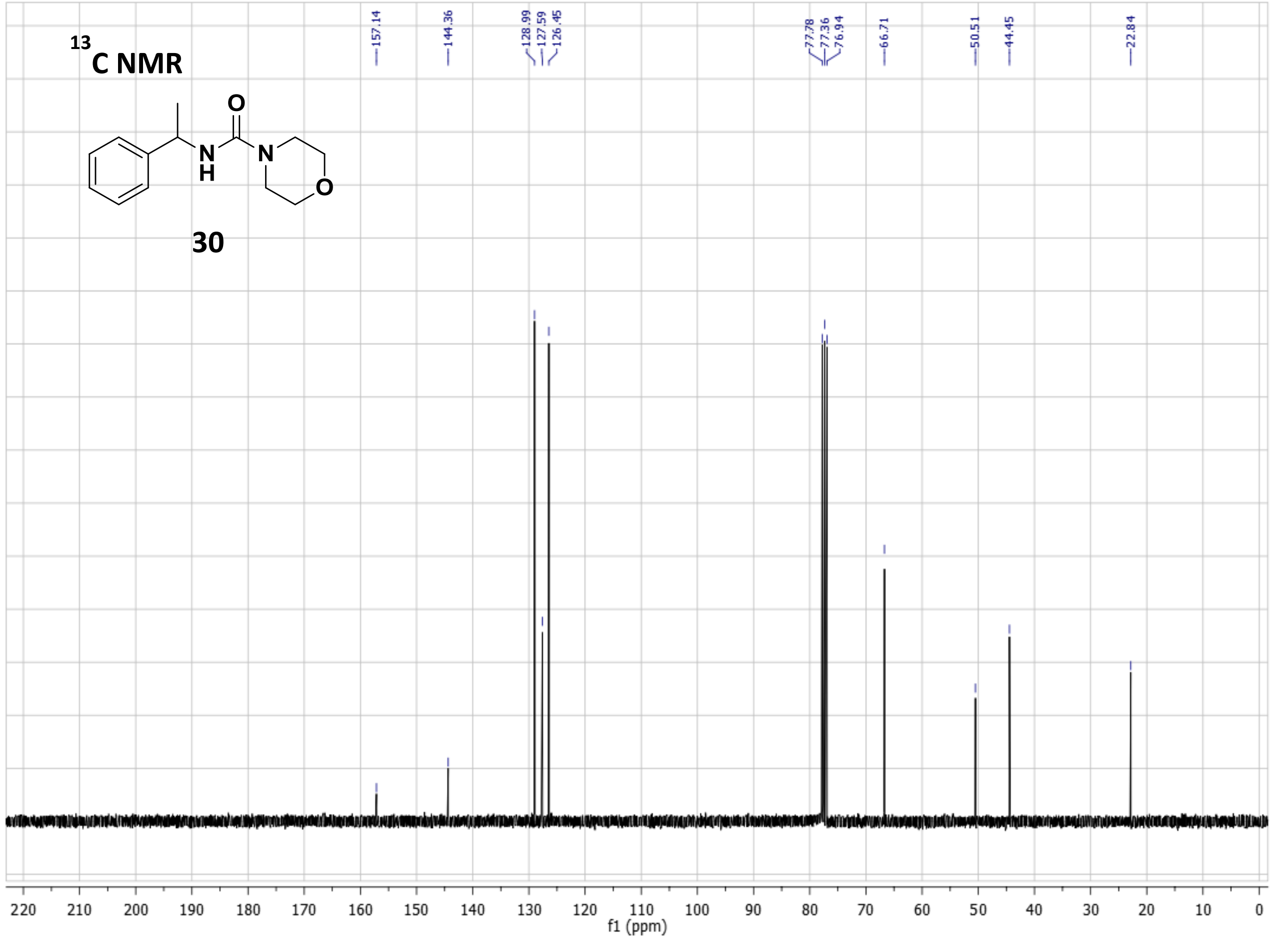

13C NMR
O
N
H
N
O
30
157.14
144.36
128.99
127.59
126.45
77.78
77.36
76.94
66.71
50.51
44.45
22.84
f1 (ppm)

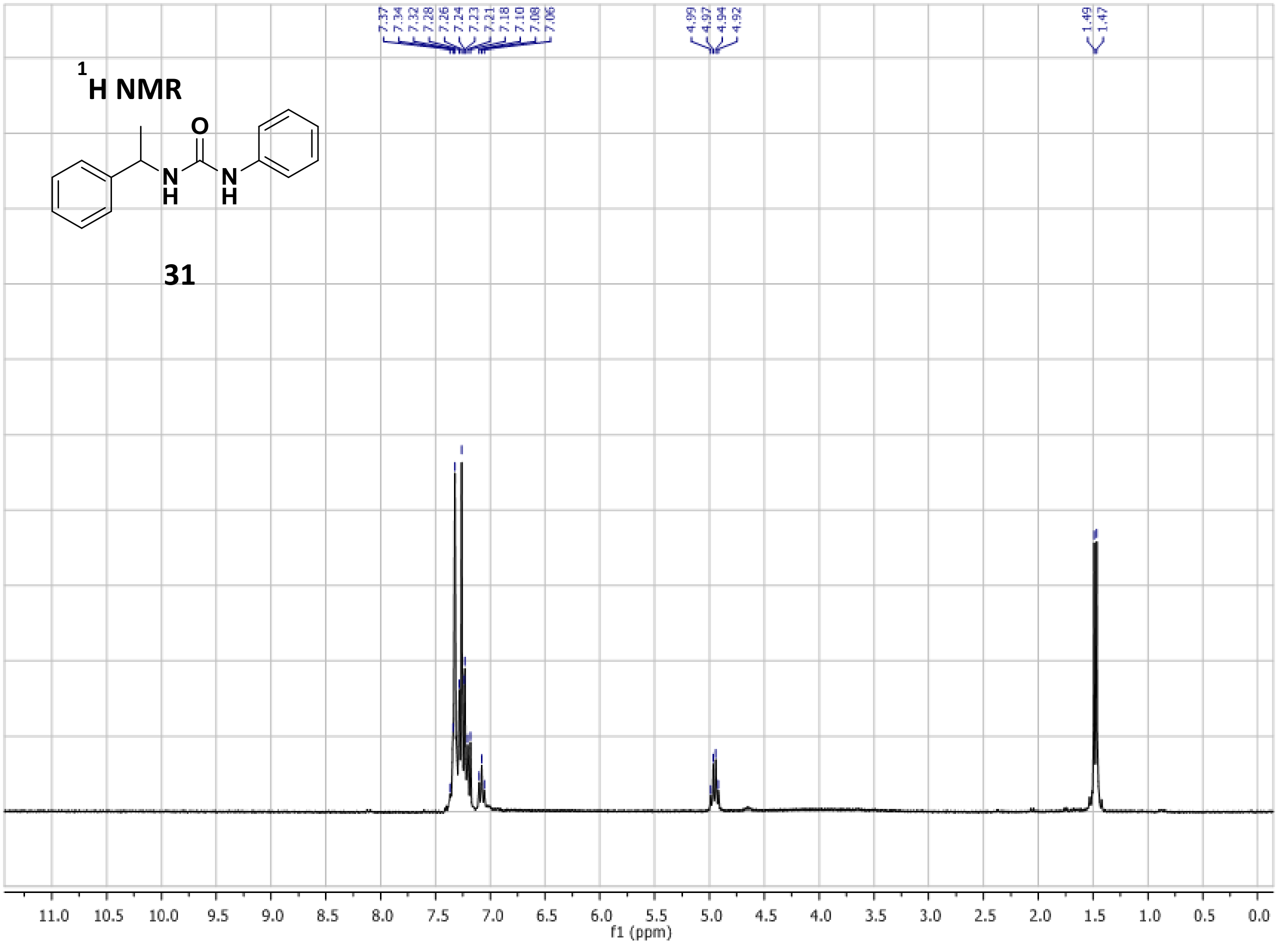
1H NMR
O
N
H
N
H
31
7.37
7.34
7.32
7.28
7.26
7.24
7.23
7.21
7.18
7.10
7.08
7.06
4.99
4.97
4.94
4.92
1.49
1.47
11.0
10.5
10.0
9.5
9.0
8.5
8.0
7.5
7.0
6.5
6.0
5.5
5.0
4.5
4.0
3.5
3.0
2.5
2.0
1.5
1.0
0.5
0.0
f1 (ppm)

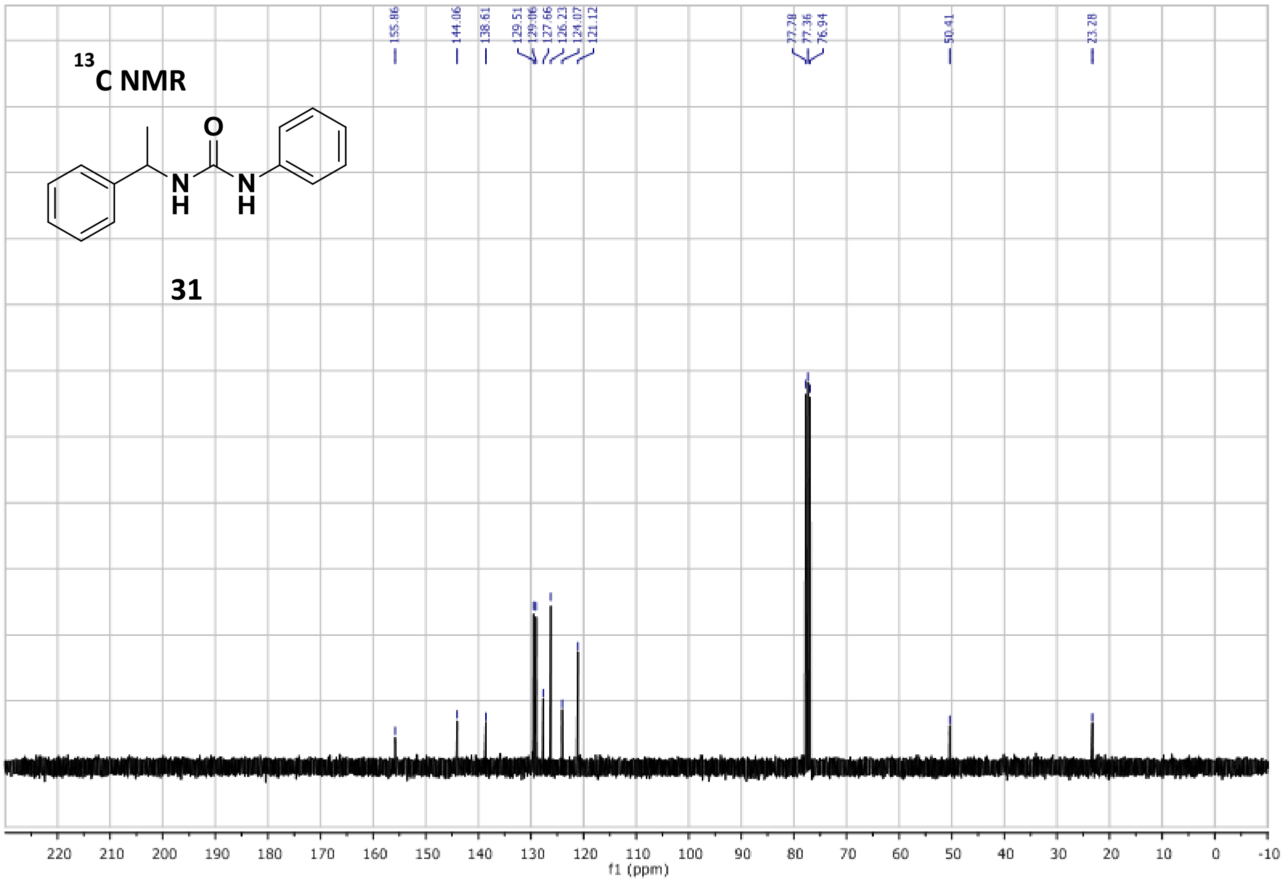
13C NMR
O
N
H
N
H
31
155.86
144.06
138.61
129.51
129.06
127.66
126.23
124.07
121.12
77.78
77.36
76.94
50.41
23.28
220
210
200
190
180
170
160
150
140
130
120
110
100
90
80
70
60
50
40
30
20
10
0
-10
f1 (ppm)

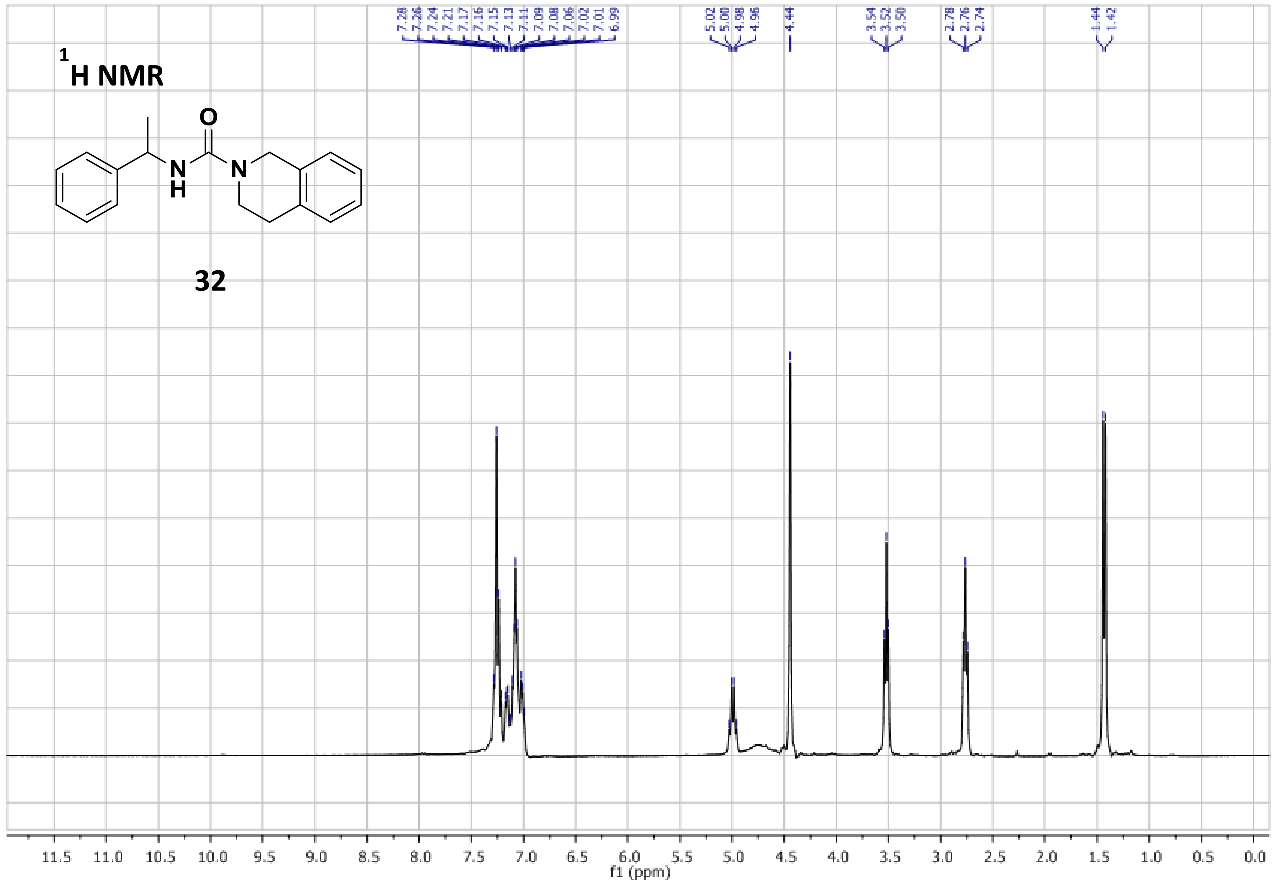
1H NMR
O
N
H
N
32
7.28 7.26 7.24 7.21 7.17 7.16 7.15 7.13 7.11 7.09 7.08 7.06 7.02 7.01 6.99
5.02 5.00 4.98 4.96
4.44
3.54 3.52 3.50
2.78 2.76 2.74
1.44 1.42
11.5 11.0 10.5 10.0 9.5 9.0 8.5 8.0 7.5 7.0 6.5 6.0 5.5 5.0 4.5 4.0 3.5 3.0 2.5 2.0 1.5 1.0 0.5 0.0
f1 (ppm)

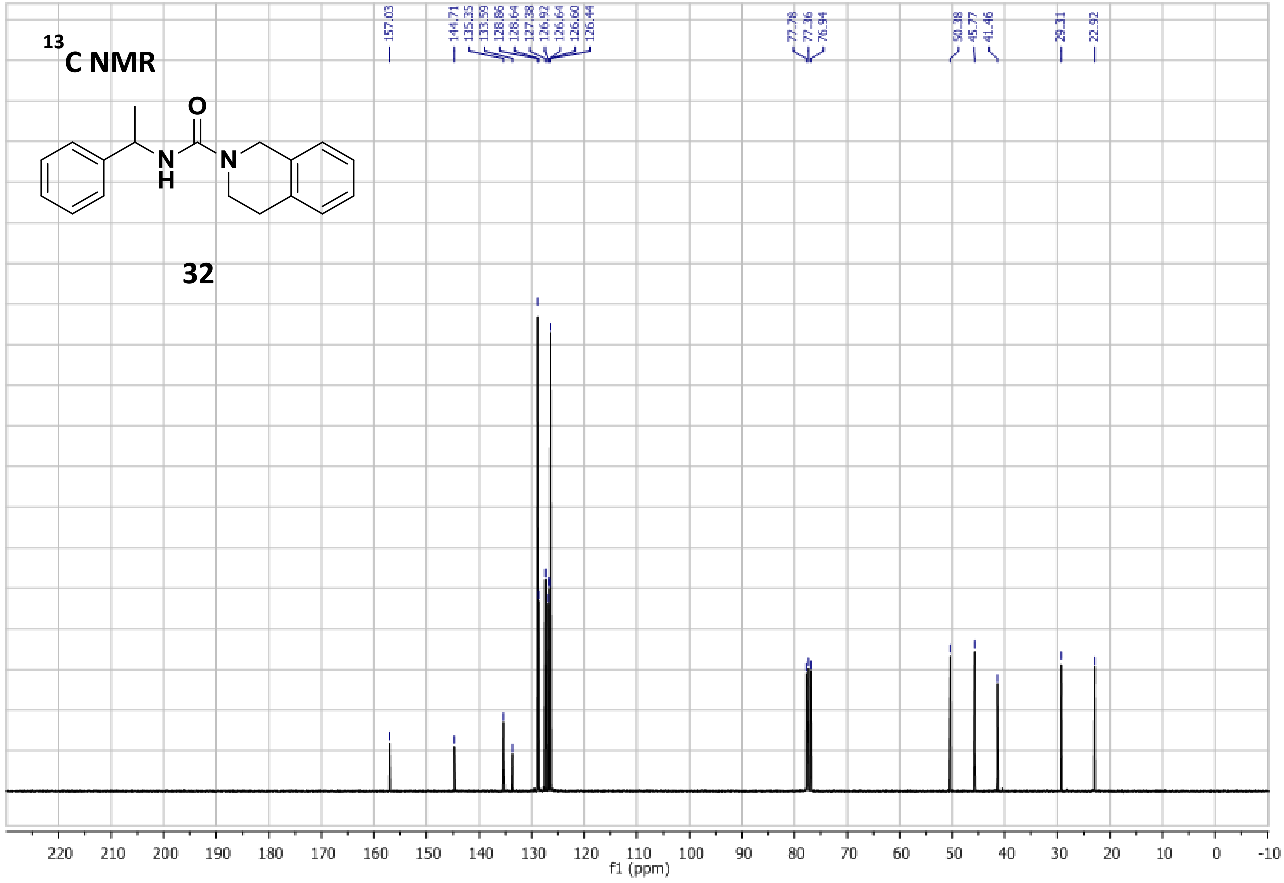
13C NMR
O
N
H
N
32
157.03
144.71 135.35 133.59 128.86 128.64 127.38 126.92 126.64 126.60 126.44
77.28 77.36 76.94
50.38 45.77 41.46
29.31
22.92
220 210 200 190 180 170 160 150 140 130 120 110 100 90 80 70 60 50 40 30 20 10 0 -10
f1 (ppm)

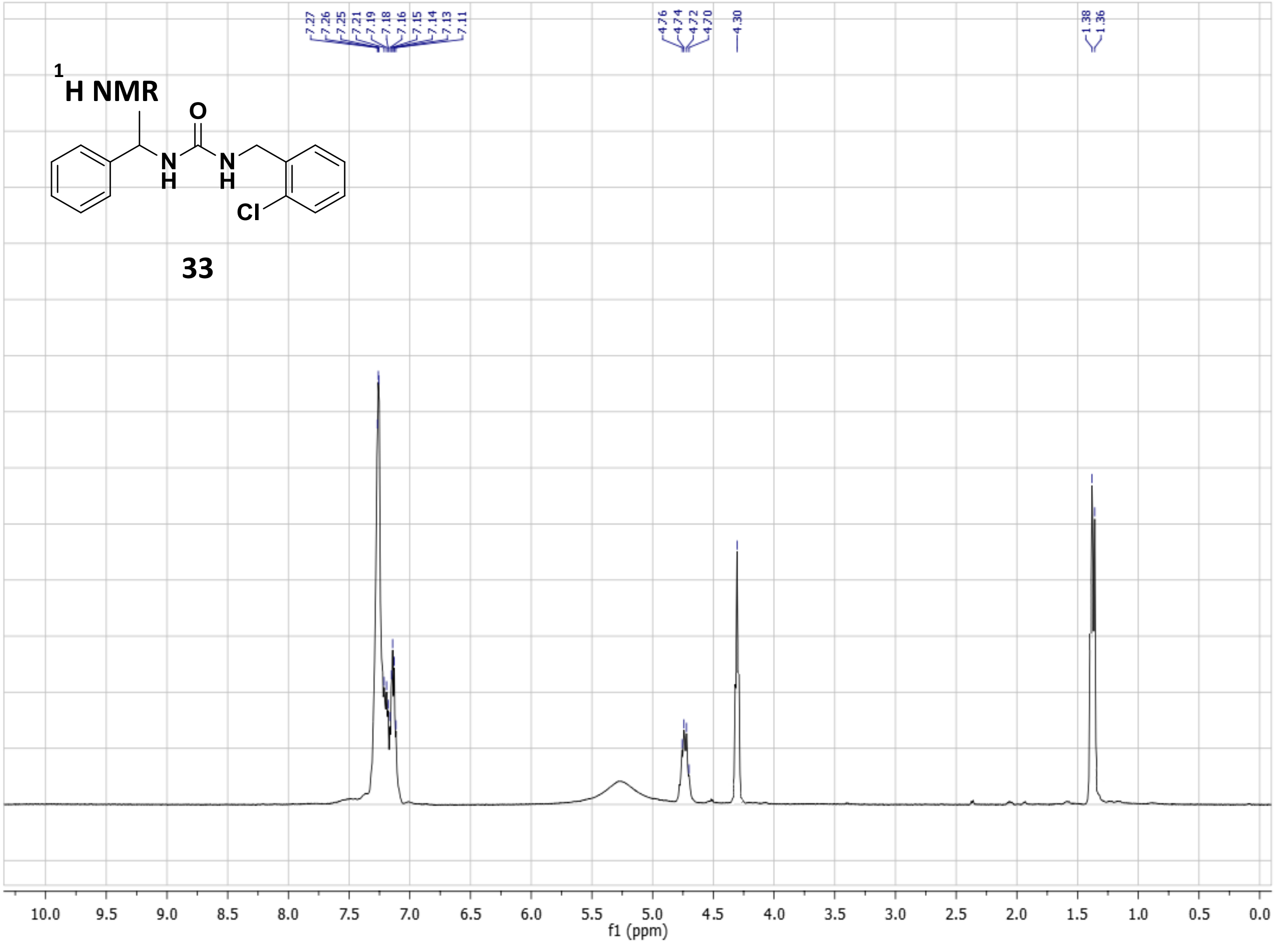
1H NMR
33
7.27 7.26 7.25 7.21 7.19 7.18 7.16 7.15 7.14 7.13 7.11
4.76 4.74 4.72 4.70
4.30
1.38 1.36
10.0 9.5 9.0 8.5 8.0 7.5 7.0 6.5 6.0 5.5 5.0 4.5 4.0 3.5 3.0 2.5 2.0 1.5 1.0 0.5 0.0
f1 (ppm)

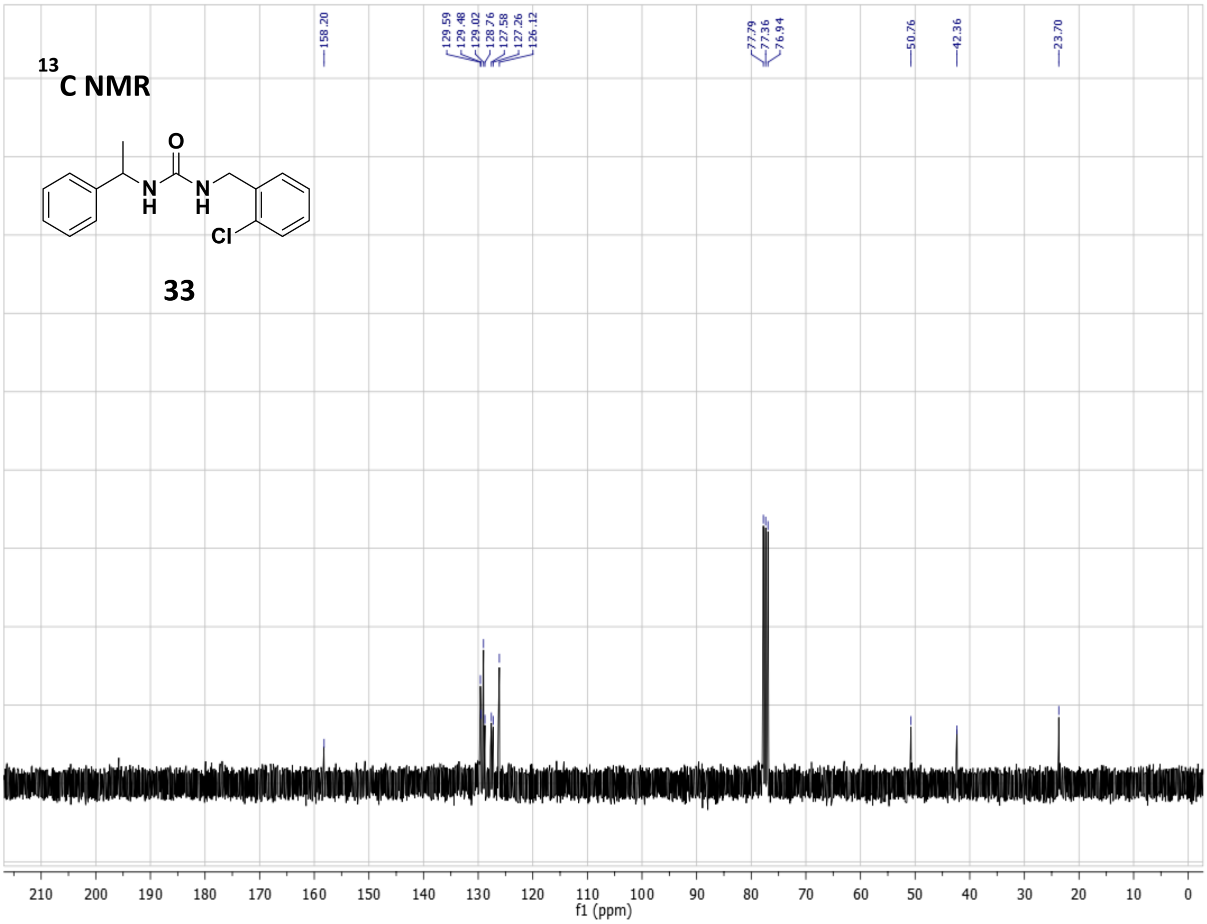

13C NMR
33
O
N
H
N
H
Cl
158.20
129.59
129.48
129.02
128.76
127.58
127.26
126.12
77.79
77.36
76.94
50.76
42.36
23.70
210 200 190 180 170 160 150 140 130 120 110 100 90 80 70 60 50 40 30 20 10 0
f1 (ppm)

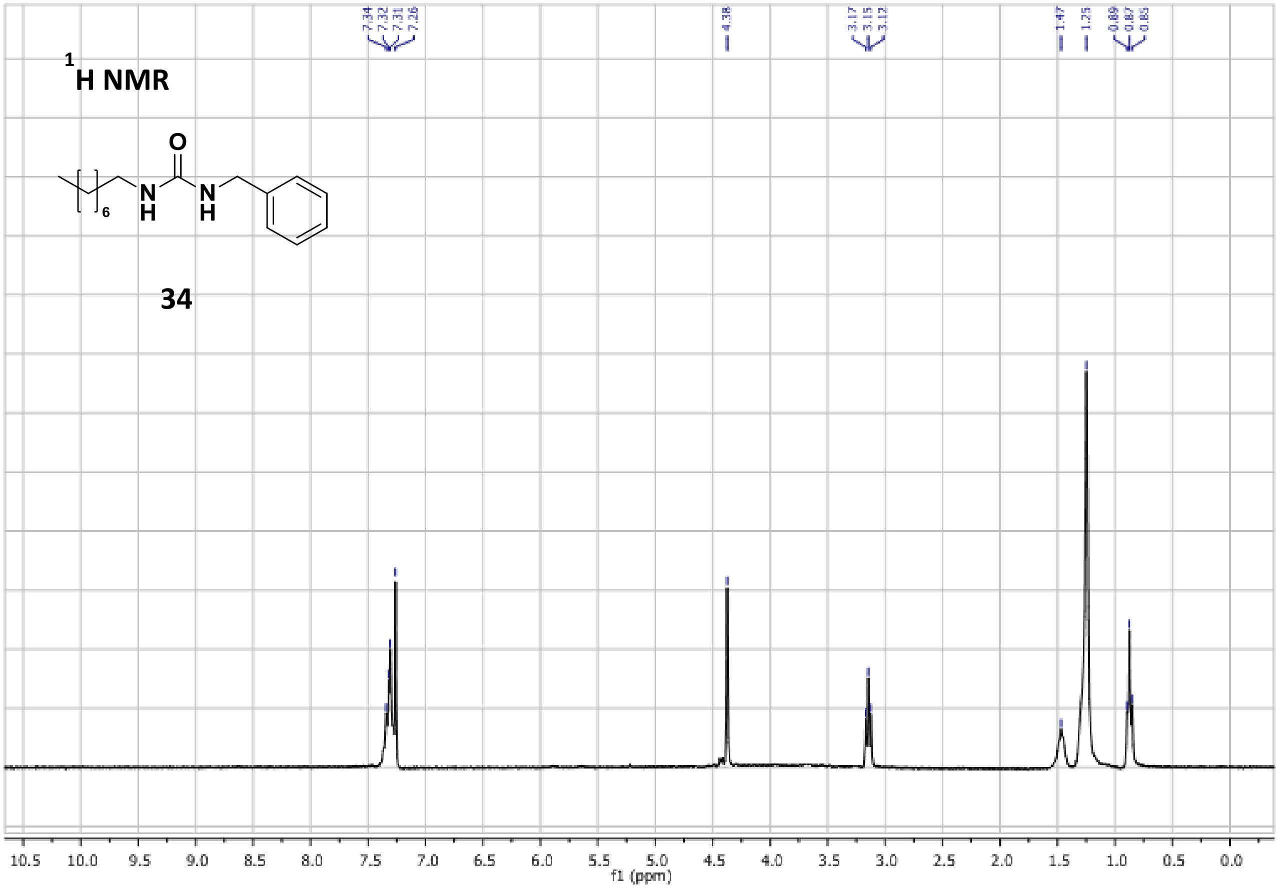

1H NMR
34
O
N
H
N
H
6
7.34
7.32
7.31
7.26
4.38
3.17
3.15
3.12
1.47
1.25
0.89
0.87
0.85
10.5 10.0 9.5 9.0 8.5 8.0 7.5 7.0 6.5 6.0 5.5 5.0 4.5 4.0 3.5 3.0 2.5 2.0 1.5 1.0 0.5 0.0
f1 (ppm)

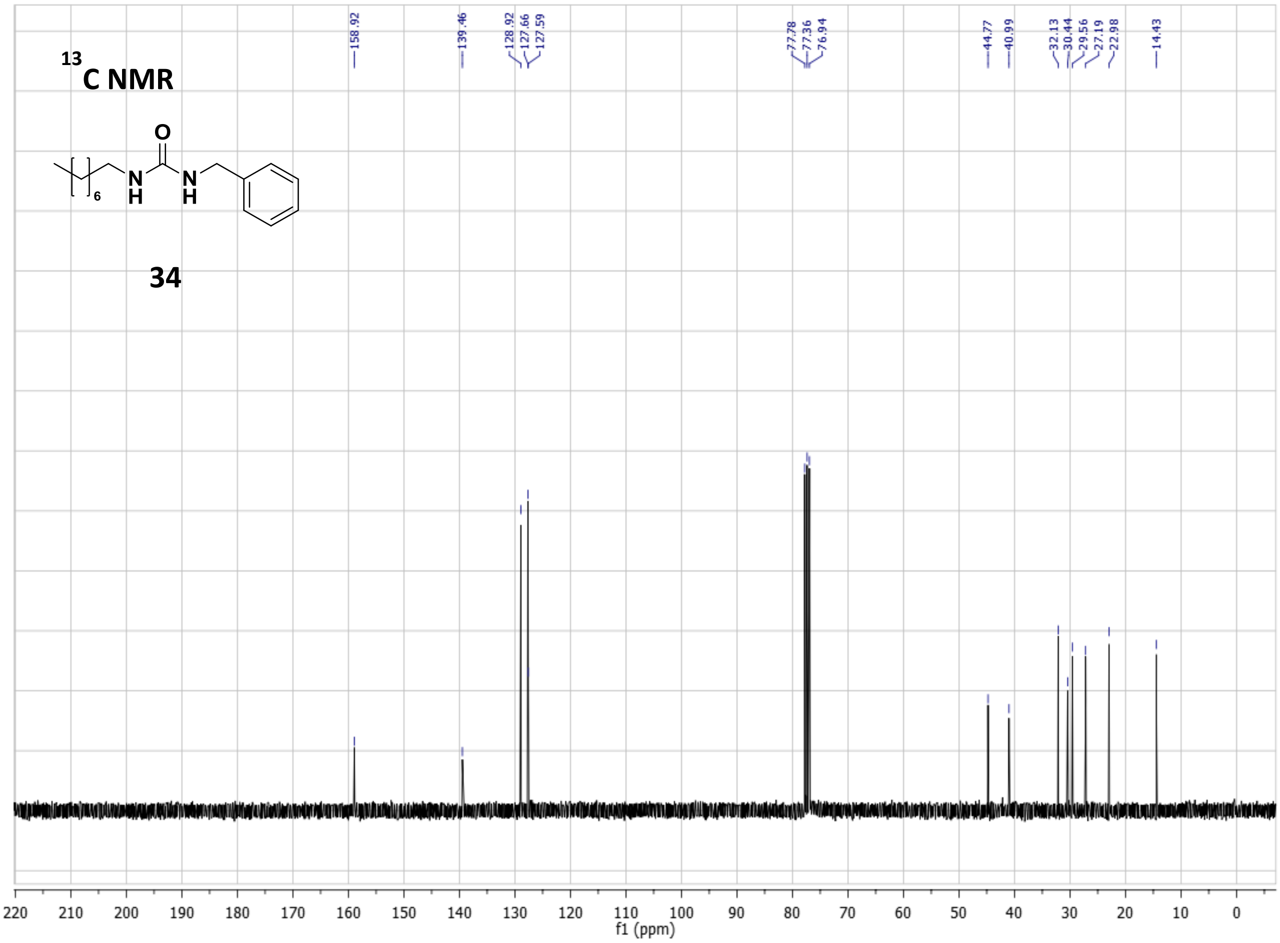
13C NMR
34
158.92
139.46
128.92
127.66
127.59
77.78
77.36
76.94
44.77
40.99
32.13
30.44
29.56
27.19
22.98
14.43
f1 (ppm)

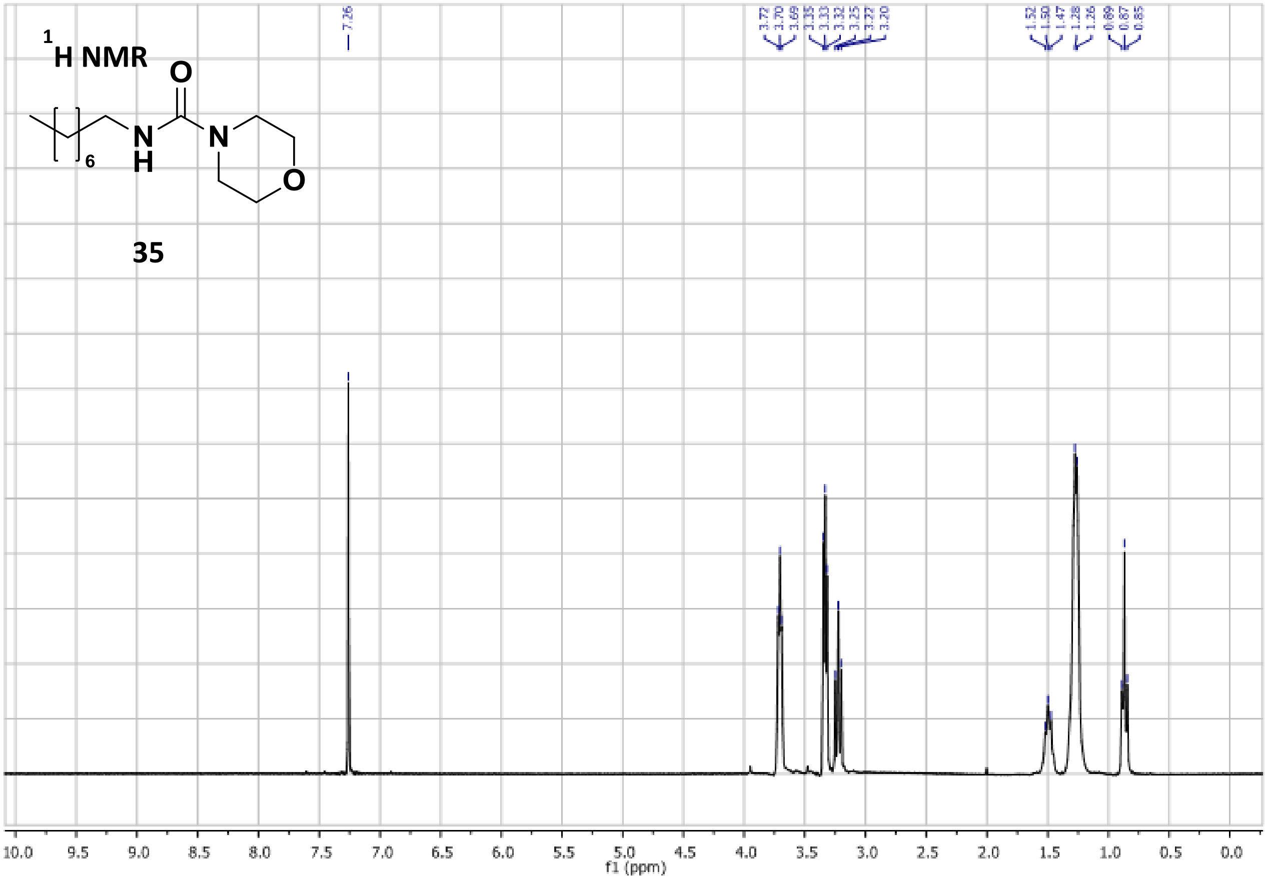
1H NMR
35
7.26
3.72
3.70
3.69
3.35
3.33
3.32
3.25
3.22
3.20
1.52
1.50
1.47
1.28
1.26
0.89
0.87
0.85
f1 (ppm)

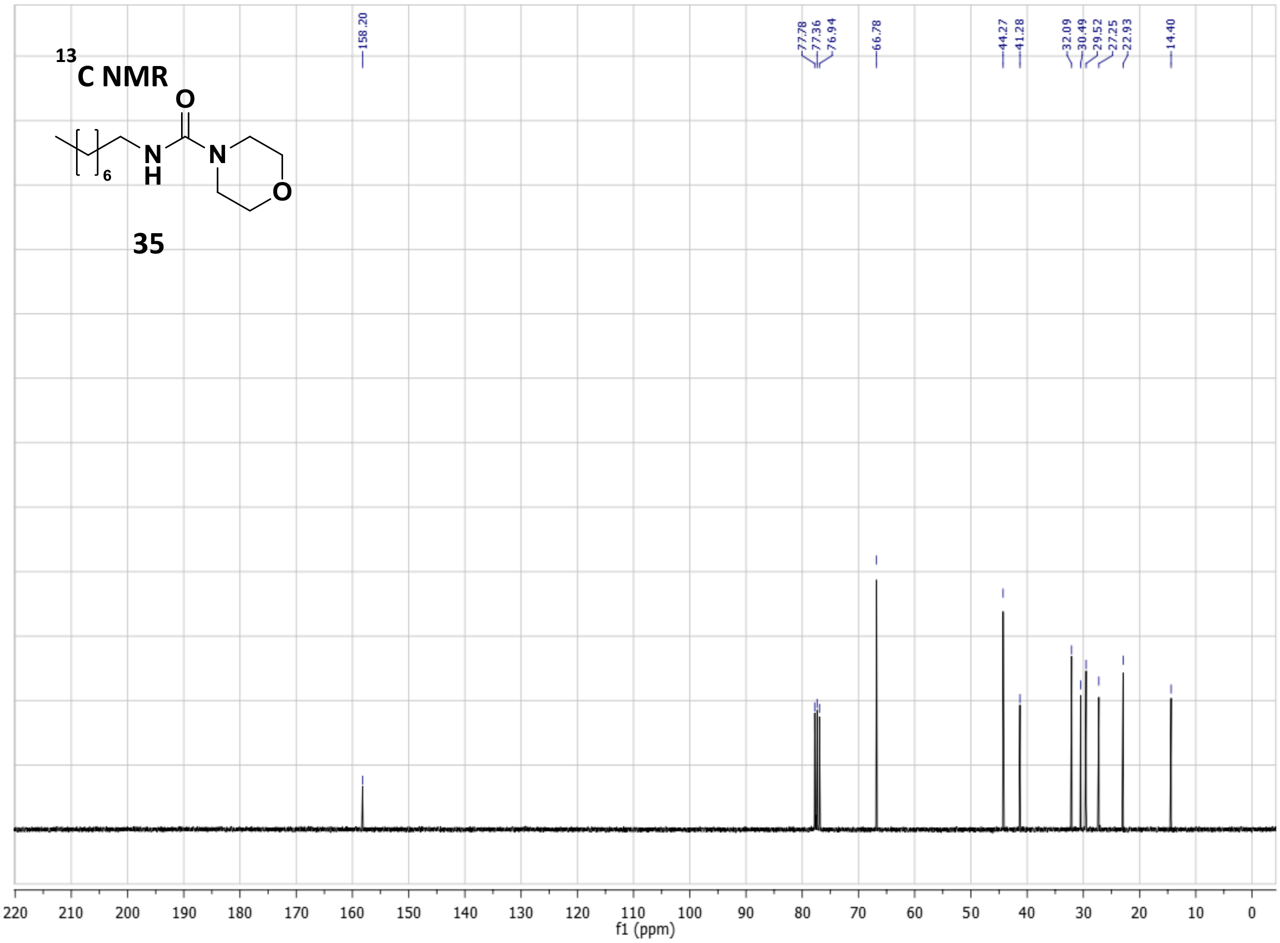

13C NMR
35
158.20
77.78
77.36
76.94
66.78
44.27
41.28
32.09
30.49
29.52
27.25
22.93
14.40
f1 (ppm)

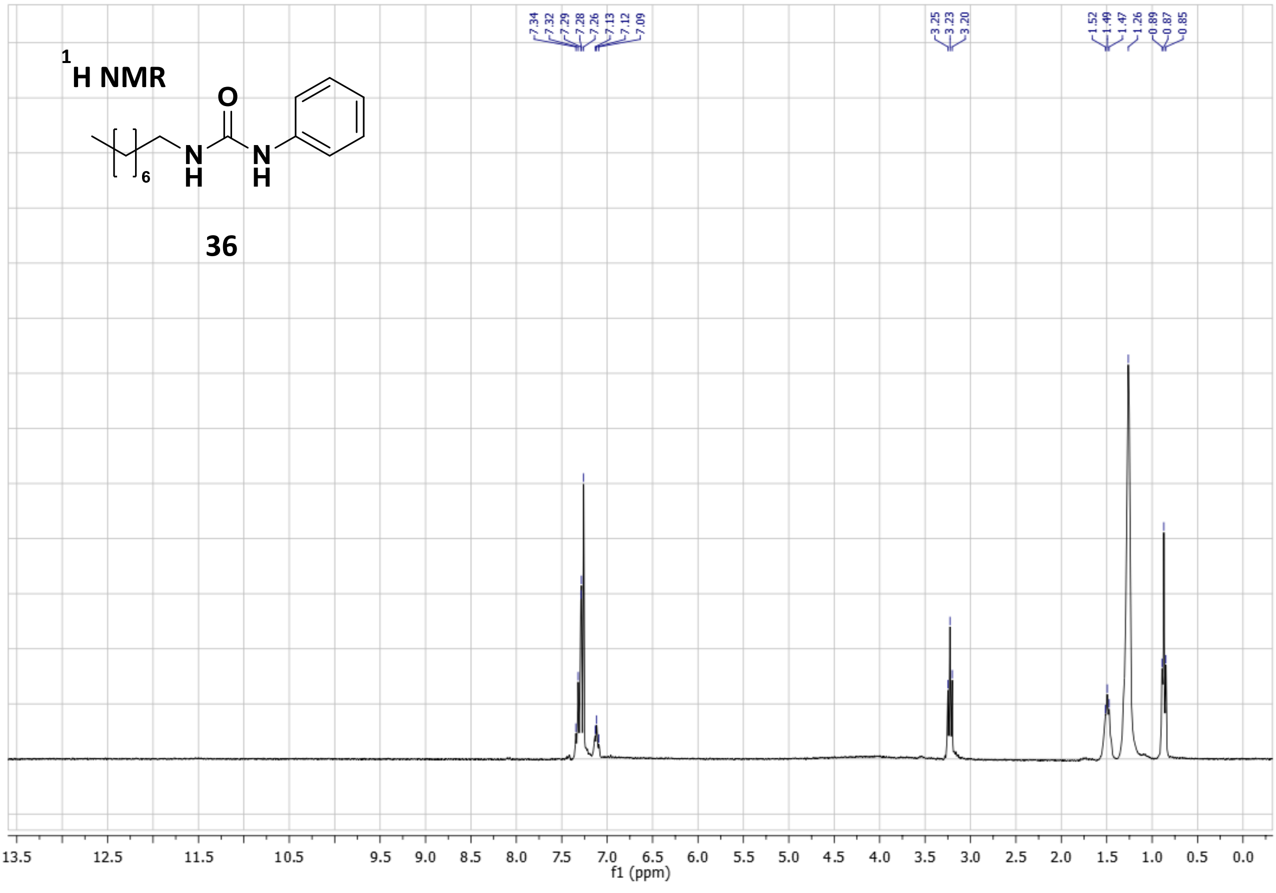

1H NMR
36
7.34
7.32
7.29
7.28
7.26
7.13
7.12
7.09
3.25
3.23
3.20
1.52
1.49
1.47
1.26
0.89
0.87
0.85
f1 (ppm)

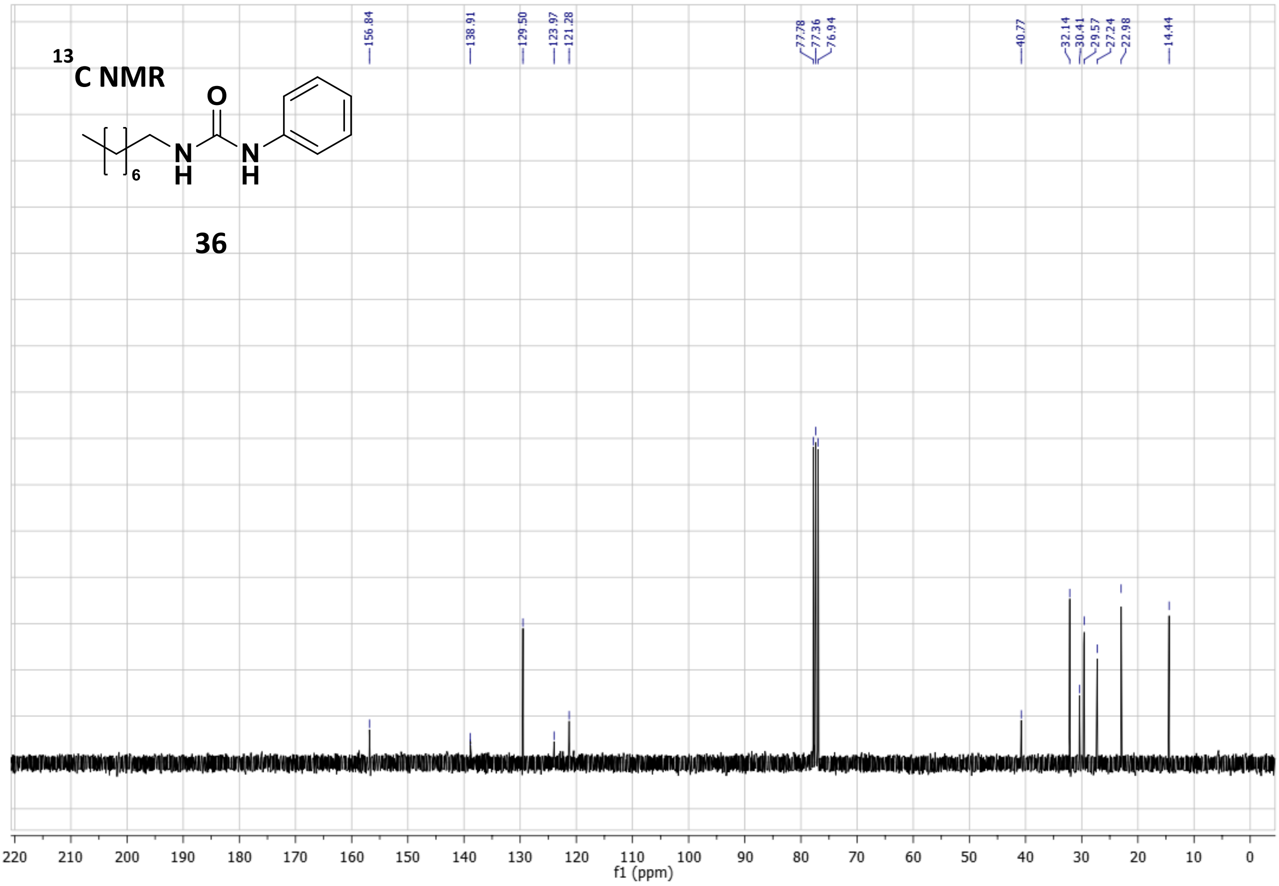

13C NMR
O
N
H
N
H
6
36
156.84
138.91
129.50
123.97
121.28
77.78
77.36
76.94
40.77
32.14
30.41
29.57
27.24
22.98
14.44
220 210 200 190 180 170 160 150 140 130 120 110 100 90 80 70 60 50 40 30 20 10 0
f1 (ppm)

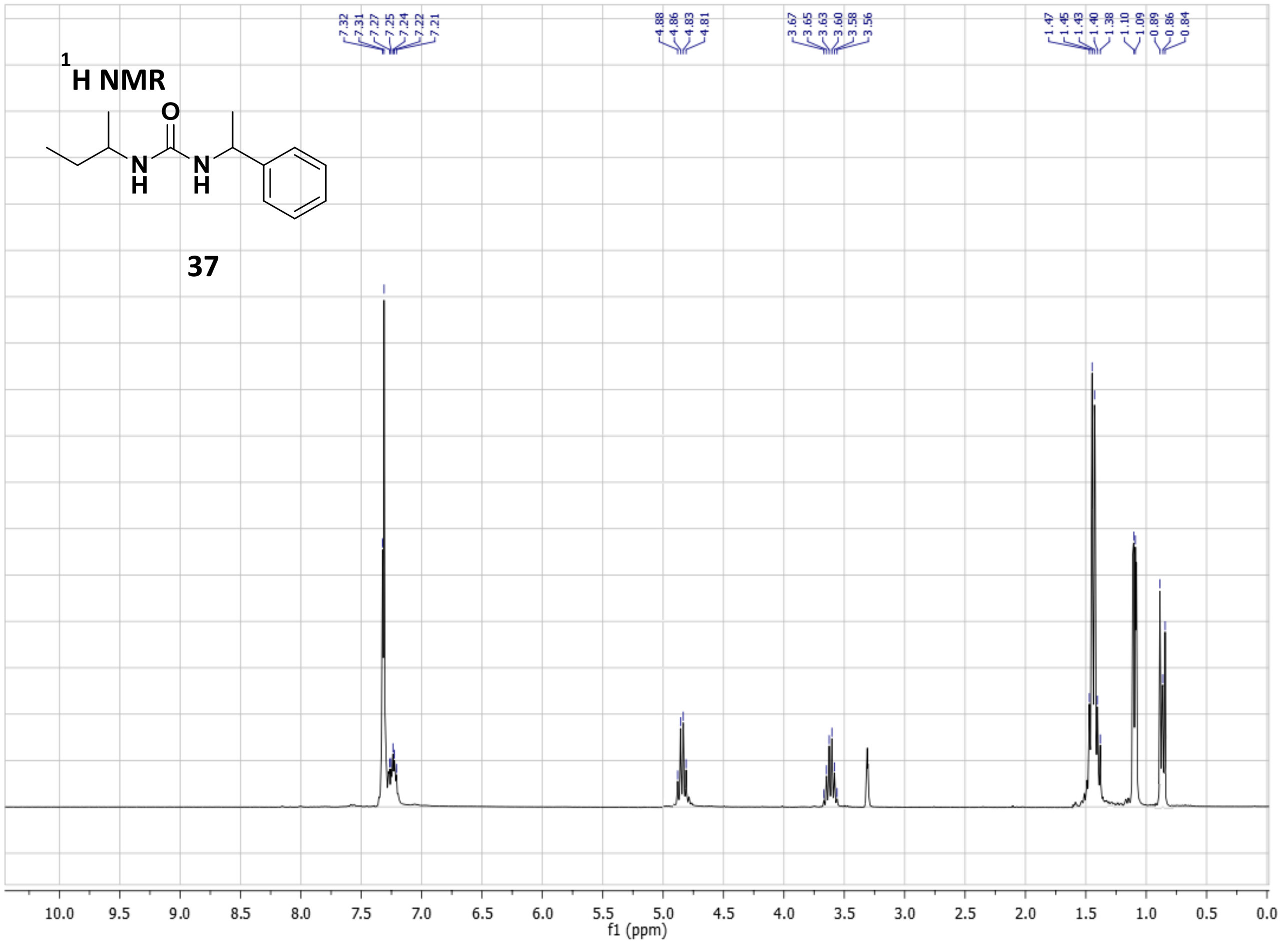

1H NMR
O
N
H
N
H
37
7.32 7.31 7.27 7.25 7.24 7.22 7.21
4.88 4.86 4.83 4.81
3.67 3.65 3.63 3.60 3.58 3.56
1.47 1.45 1.43 1.40 1.38 1.10 1.09 0.89 0.86 0.84
10.0 9.5 9.0 8.5 8.0 7.5 7.0 6.5 6.0 5.5 5.0 4.5 4.0 3.5 3.0 2.5 2.0 1.5 1.0 0.5 0.0
f1 (ppm)

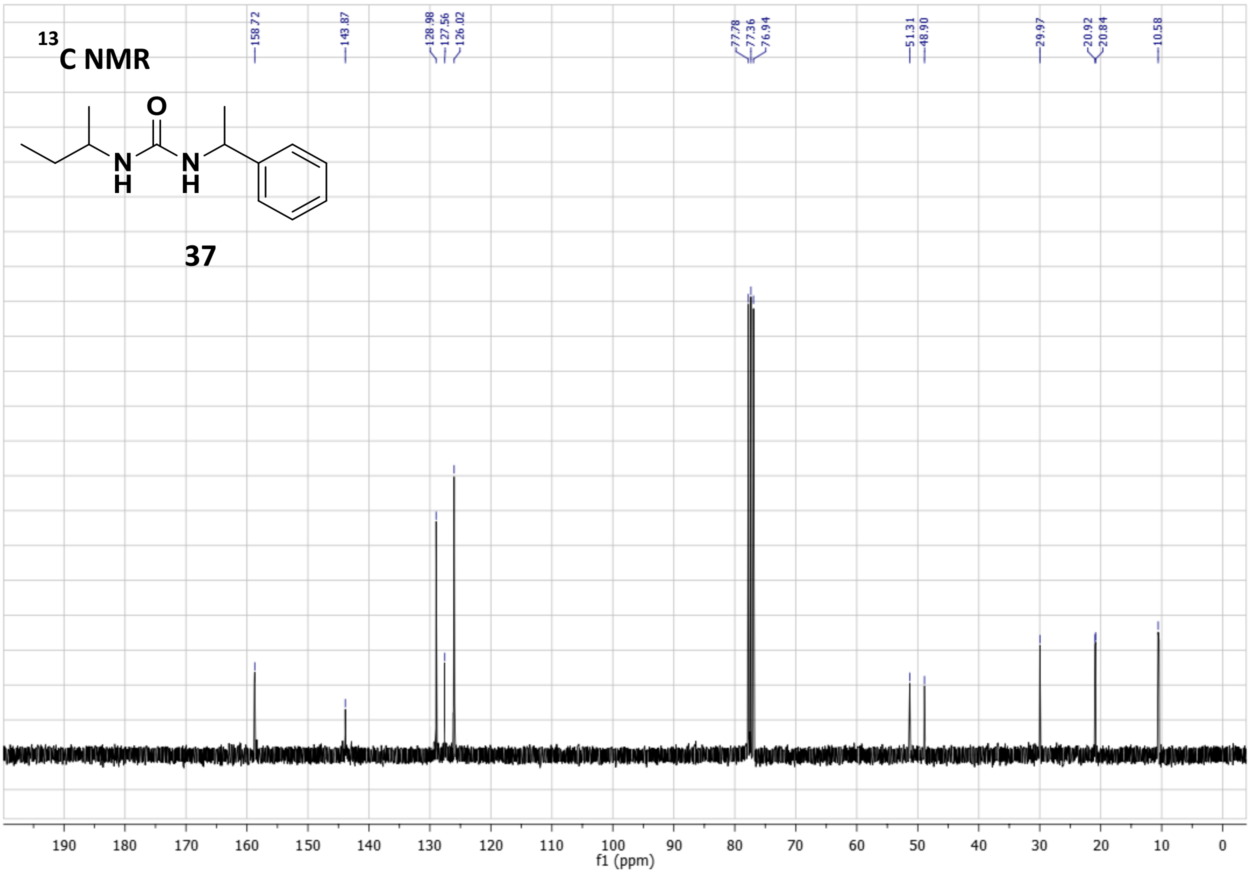

13C NMR
O
N
H
N
H
37
158.72
143.87
128.98 127.56 126.02
77.78 77.36 76.94
51.31 48.90
29.97
20.92 20.84
10.58
190 180 170 160 150 140 130 120 110 100 90 80 70 60 50 40 30 20 10 0
f1 (ppm)

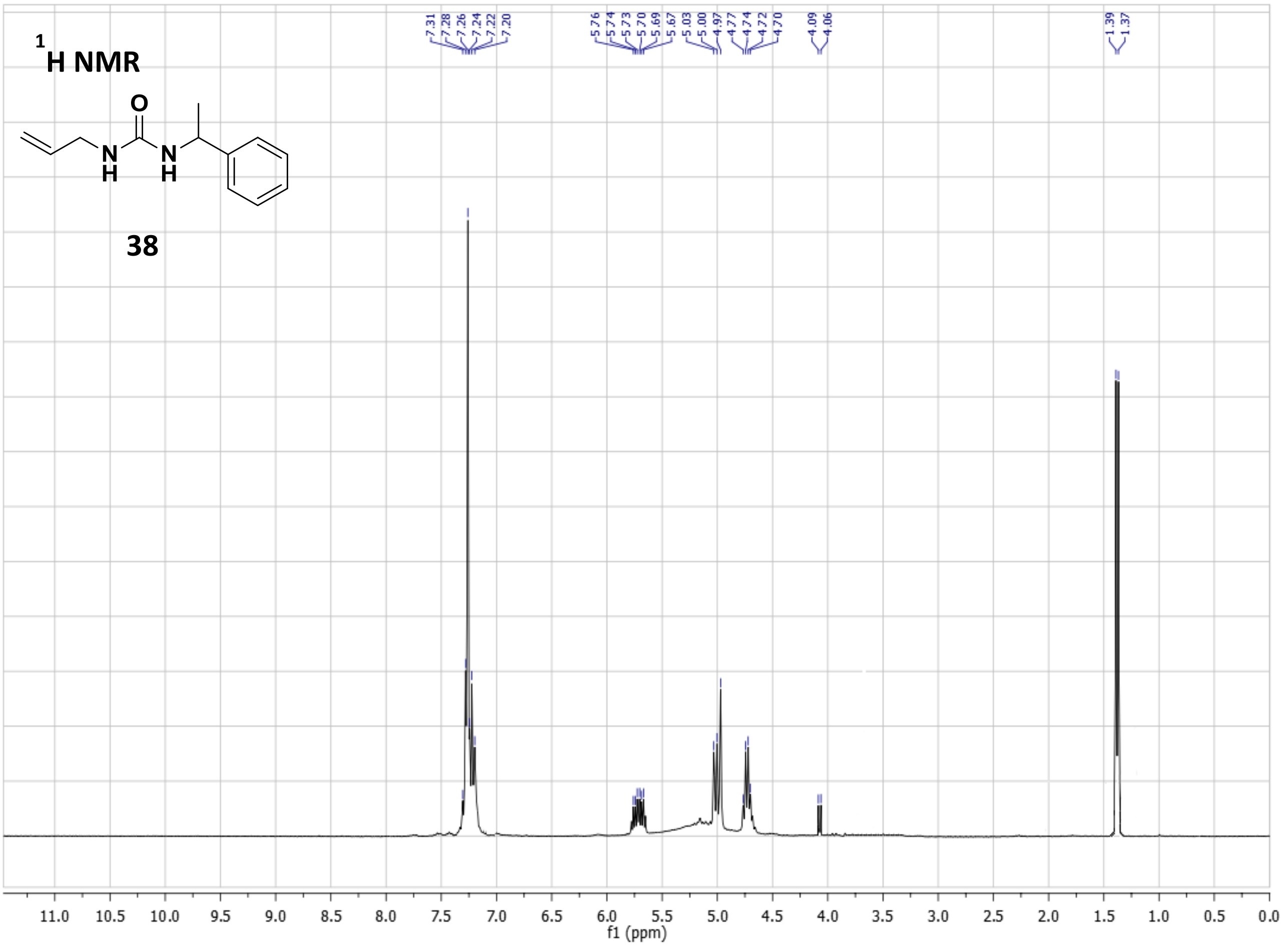

1H NMR
O
N
H
N
H
38
7.31
7.28
7.26
7.24
7.22
7.20
5.76
5.74
5.73
5.70
5.69
5.67
5.03
5.00
4.97
4.77
4.74
4.72
4.70
4.09
4.06
1.39
1.37
11.0 10.5 10.0 9.5 9.0 8.5 8.0 7.5 7.0 6.5 6.0 5.5 5.0 4.5 4.0 3.5 3.0 2.5 2.0 1.5 1.0 0.5 0.0
f1 (ppm)

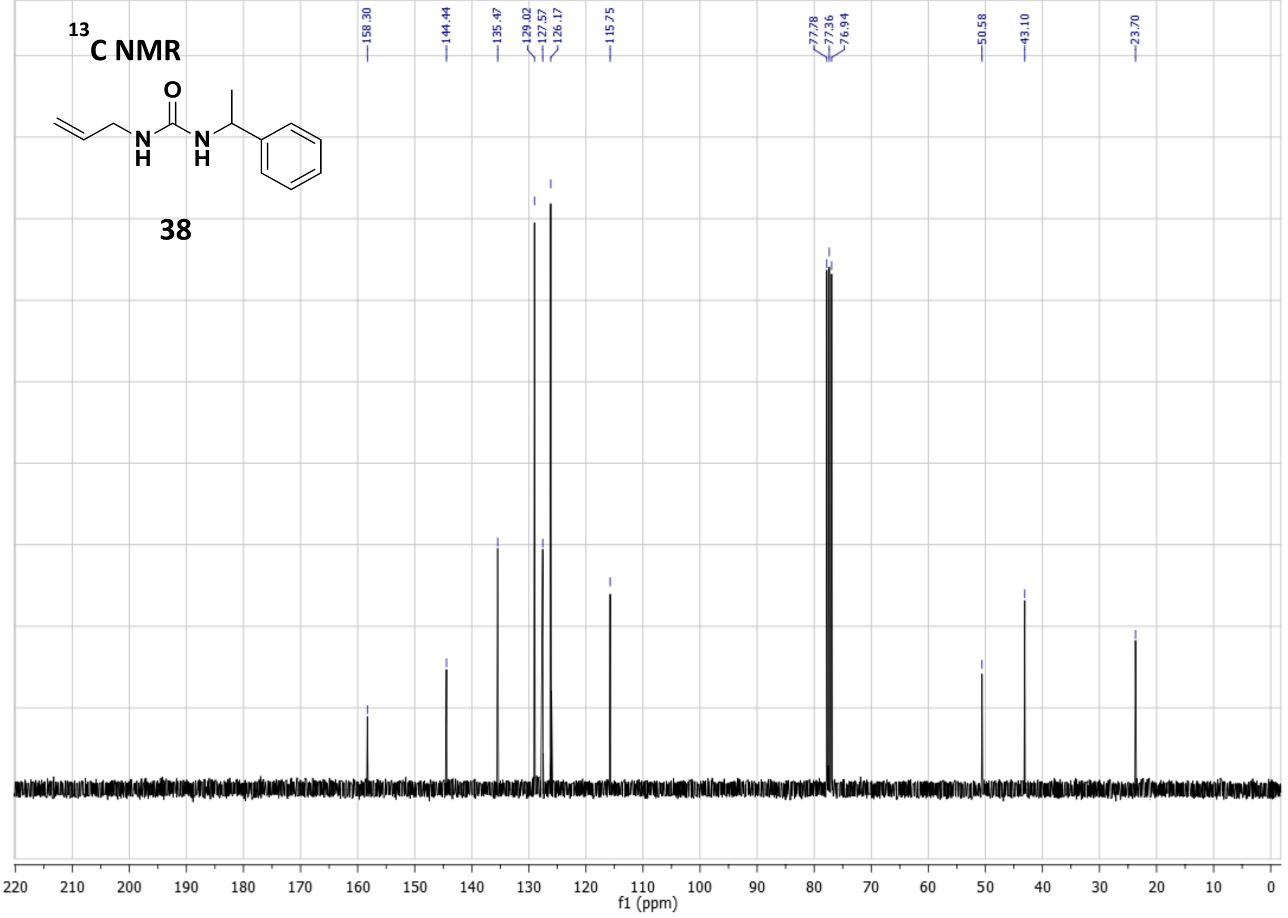

13C NMR
O
N
H
N
H
38
158.30
144.44
135.47
129.02
127.57
126.17
115.75
77.78
77.36
76.94
50.58
43.10
23.70
220 210 200 190 180 170 160 150 140 130 120 110 100 90 80 70 60 50 40 30 20 10 0
f1 (ppm)

References

1. Tsopmo, A.; Ngnokam, D.; Ngamga, D.; Ayafor, J. F.; Sterner, O. *J. Nat. Prod.* **1999,** *62*, 1435–1436.
2. Park, J. H.; Yoon, J. C.; Chunga, Y. K. *Adv. Synth. Catal.* **2009,** *351*, 1233–1237.
3. Dube, P.; Fine Nathel, N. F.; Vetelino, M.; Couturier, M.; Larrivee Aboussafy, C.; Pichette, S.; Jorgensen, M. L.; Hardink M. *Org. Lett.* **2009,** *11*, 5622–5625.
4. Peterson, S. L.; Stucka, S. M.; Dinsmore C. J. *Org. Lett.* **2010**, *12*, 1340–1343.
5. Perrone, M. G.; Bleve, L.; Santandrea, E.; Vitale, P.; Niso, M.; Scilimati, A. *ChemMedChem* **2009,** *4*, 2080–2097.
